# Crystal structure of a β1,3-Glucosyltransferase reveals an unusual substrate recognition by a two-domain GT-A fold glycosyltransferase

**DOI:** 10.1016/j.jbc.2026.113418

**Published:** 2026-08-10

**Authors:** Steven J. Berardinelli, Renuka Kadirvelraj, Kelvin B. Luther, Zhongwei Gao, Digantkumar Chapla, Chin Huang, Daniel M. Tehrani, Ao Zhang, Kelley W. Moremen, Zachary A. Wood, Robert S. Haltiwanger

**Affiliations:** 1 Complex Carbohydrate Resource Center, Department of Biochemistry and Molecular Biology, University of Georgia, Athens, Georgia, USA; 2 Department of Biochemistry & Molecular Biology, University of Georgia, Athens, Georgia, USA

**Keywords:** *O*-fucosylation, glycosyltransferase, Peters plus Syndrome, thrombospondin type 1 repeats, X-ray crystallography

## Abstract

β1,3-Glucosyltransferase (B3GLCT) adds a glucose onto *O*-linked fucose on thrombospondin type 1 repeats (TSRs). Protein *O*-fucosyltransferase 2 (POFUT2) first transfers a fucose to properly folded TSRs containing a consensus sequence for *O*-fucosylation. This uncommon *O*-fucose modification is then extended to a glucose-fucose disaccharide by B3GLCT. B3GLCT is a GT-A fold glycosyltransferase and pathogenic variants cause Peters Plus Syndrome (PTRPLS, OMIM #261540), a Congenital Disorder of Glycosylation (CDG). Most GT-A fold family members have a single GT-A domain, but B3GLCT contains an additional GT-A domain. To assess the function of the additional GT-A domain and binding of TSR substrates, we determined the crystal structure of an *O*-fucosylated TSR (Fuc-*O*-TSR3) from Thrombospondin-1 bound to B3GLCT. The additional GT-A domain is essential for substrate binding but is catalytically inactive: it does not bind UDP or Mn^2+^ and is not a β1,3-glucosyltransferase. It also creates a deep pocket with a vestigial active site located on the opposite face of the Fuc-*O*-TSR3 substrate binding site. The Fuc-*O*-TSR3 acceptor substrate binds in a cleft between the two GT-A domains, each of which has evolved hypervariable regions for Fuc-*O*-TSR recognition. This structure is an unusual example of a glycosyltransferase with a catalytically inactive extra GT-A domain, providing insight into the binding of B3GLCT’s diverse Fuc-*O*-TSR substrates. We also discuss how B3GLCT mimics the two-domain structure of GT-B fold glycosyltransferases. All GT-B fold glycosyltransferases contain two bilobal Rossmann-like fold domains, like B3GLCT. Our data also explain how PTRPLS-associated variants and predicted pathogenic mutations disrupt B3GLCT function.

*O*-fucosylation is an unusual protein modification that occurs on properly folded, cysteine-rich domains. Four protein *O*-fucosyltransferases (POFUT1-4) are known, each acting on specific protein domain types (1). POFUT2 exclusively modifies Thrombospondin Type 1 Repeat (TSRs) domains (2). In TSRs, the initial *O*-fucose modification can be further extended in the endoplasmic reticulum (ER) by B3GLCT, an *O*-fucose β1,3 glucosyltransferase, producing the Glc-β1,3-Fuc-α1-*O*-TSR glycan (Fig. 1, *A*–*C*) (3, 4). All TSRs contain 6 highly conserved cysteines which form three disulfide bonds. For *O*-fucosylation to occur, the TSRs must be correctly folded and contain the consensus sequence [C^1^-X-X-(S/T)-C^2^] between the first and second cysteines (Group 1 TSRs) or [C^2^-X-X-(S/T)-C^3^] between the second and third cysteines (Group 2 TSRs) (1). The modification occurs on the hydroxyl group of the Ser/Thr side chains within this motif. In the human genome, there are 49 proteins containing TSRs with the consensus sequence, and most possess multiple TSRs arranged in tandem repeats (1).

**Figure 1:**
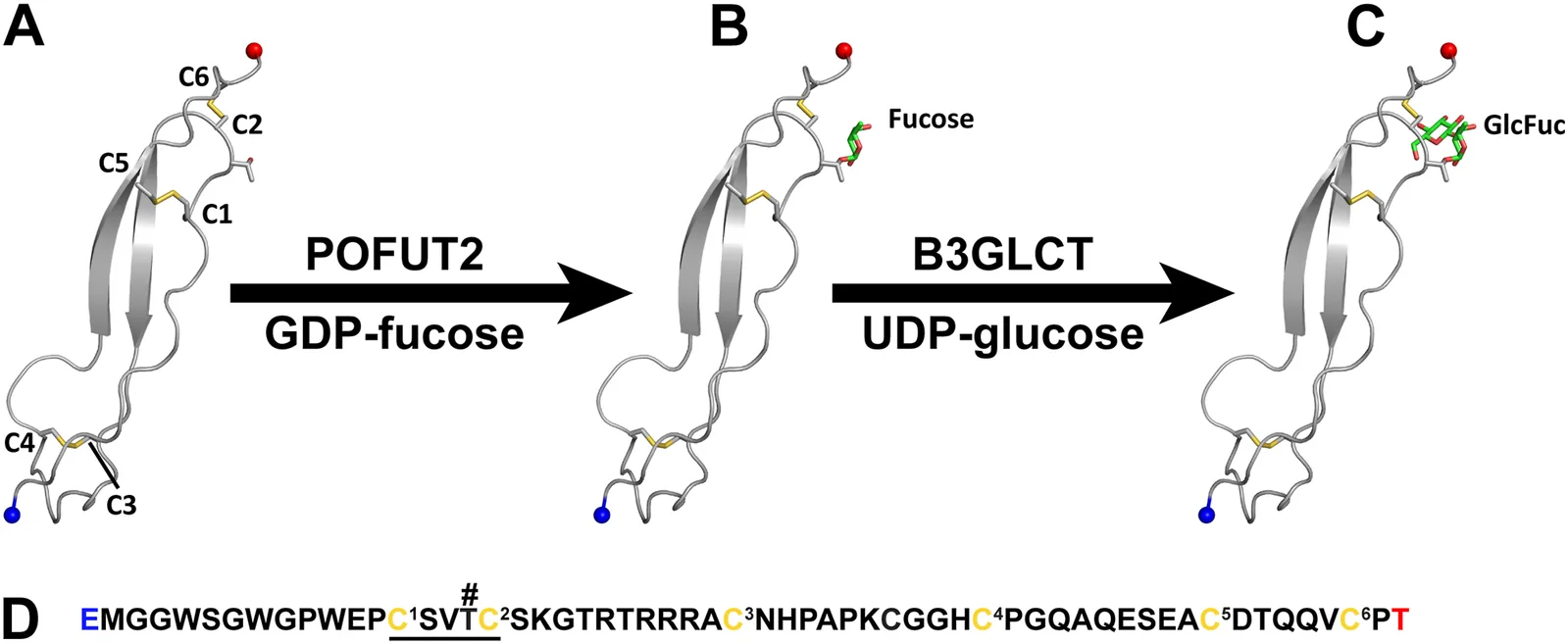
**Cartoon depiction of the TSR *O*-fucosylation pathway.***A*, unmodified TSRs with the consensus sequence for *O*-fucosylation between cysteine 1 and cysteine 2 [C^1^-X-X-(S/T)-C^2^] for Group 1 TSRs, and between cysteine 2 and cysteine 3 [C^2^-X-X-(S/T)-C^3^] for Group 2 TSRs, are modified by POFUT2 and GDP-fucose. All six conserved cysteines are labeled as C1 – C6. *Blue* spheres indicate the N-terminus of the TSR. *Red* spheres indicate the C-terminus on the TSR. *B*, POFUT2 transfers a fucose to the TSR from GDP-fucose. *C*, B3GLCT transfers a glucose to the *O*-fucose to generate the Glucose-Fucose disaccharide. *D*, the amino acid sequence of the crystal structure of TSR2 from human properdin is depicted in in panels (*A*-*C*) (pdb = 6RUS) (53). The consensus sequence for the TSR depicted is underlined. The modified Thr residue in the consensus sequence is indicated with a hashtag. Cysteines are in mustard *yellow*. The N- and C-terminal residues are colored blue and red respectively.

TSR *O*-fucosylation stabilizes TSRs in their 3-dimensional fold, thereby facilitating protein maturation and secretion to the extracellular matrix (ECM) (5, 6, 7). Since POFUT2 and B3GLCT only recognize and modify properly folded TSRs, this modification has been proposed to function as a non-canonical quality control pathway for TSRs in the endoplasmic reticulum (ER) (5, 6). Beyond its role in folding and quality control, TSR *O*-fucosylation can also mediate extracellular interactions. For example, an *O*-fucosylated glycan on a TSR within a BAI-adhesion GPCR is essential for binding to the RTN4/NoGo-receptor (8). TSR *O*-fucosylation is also essential for metazoan survival: knockout of *Pofut2* in mouse models is embryonic lethal, causing severe gastrulation defects (9). Furthermore, pathogenic variants in *B3GLCT* cause Peters Plus Syndrome (PTRPLS, OMIM #261540), a Congenital Disorder of Glycosylation (CDG) characterized by eye and bone-growth phenotypes (10, 11, 12, 13, 14). Knockout of *B3glct* in mice phenocopies many of the bone-growth defects (15). TSRs and *O*-fucosylation have also been studied in the context of malaria transmission further advancing the research of *O*-fucosylation outside of metazoans and humans (16, 17, 18).

B3GLCT is a GT-A fold glycosyltransferase (GT) that belongs to the Carbohydrate-Active enZYmes Database (CAZy) GT-31 family, one of the largest GT families in the CAZy database (https://www.cazy.org) (19). Notably, B3GLCT contains two GT-A fold domains (Fig. S1*A*), whereas most GT-A fold enzymes typically have a single GT-A domain with one active site. A small number of GT-A fold enzymes contain more than one GT-A domain (Table S1); in those cases, each domain generally exhibits distinct glycosyltransferase activities, utilizing different nucleotide-sugar donors and different acceptor substrates (20). In contrast, B3GLCT is unusual in that it acts on a single type of substrate (*O*-fucosylated TSRs) and its additional GT-A domain appears to be catalytically inactive (13).

GT-A fold GTs share several conserved structural features. These include a Rossman-like fold for nucleotide sugar-donor binding, a DxD motif that mediates nucleotide sugar-donor binding and metal coordination, a glycine-rich loop (G-loop), an xED motif containing the catalytic base for enzymatic activity, and a *C*-terminal histidine residue critical for metal coordination in the active site (21). In addition, GT-A enzymes contain three hypervariable regions (HV-1, -2, and -3) that contribute to nucleotide sugar-donor and acceptor substrate binding (21, 22).

Using a crystal structure of B3GLCT bound to the third TSR from thrombospondin 1 modified with *O*-fucose (Fuc-*O*-TSR3), we show that substrate binding is mediated primarily by the HV-2 region of each GT-A domain as well as by a loop between the α4-α5 helices in each domain. The HV-2 regions of B3GLCT have evolved to enable selective recognition of Fuc-*O*-TSRs while maintaining enough variability to accommodate a wide range of *O*-fucosylated TSRs from different proteins. Our structural analysis further suggests that the N-terminal GT-A domain of B3GLCT is incapable of binding UDP or Mn^2+^. In addition, we provide evidence that the N-terminal GT-A domain has a vestigial active site located in a deep pocket that is formed by both domains of B3GLCT. This deep pocket is located on the opposite face of B3GLCT relative to the Fuc-*O*-TSR3 substrate binding site. We therefore propose that the principal function of the N-terminal domain is to bind and dock *O*-fucosylated TSR substrates, rather than to catalyze glycosyltransferase activity. In addition, we also propose that B3GLCT is a hybrid of a GT-A fold and a GT-B fold glycosyltransferase by mimicking the bilobal, two Rossmann-like domain structure of GT-B fold GTs in which the N-terminal domain plays a role in acceptor binding while the primary function of the C-terminal domain is catalysis of monosaccharide transfer to the acceptor.

## Results

### The crystal structure of the B3GLCT:UDP-Mn^2+^:Fuc-O-TSR3 complex

**Figure 2:**
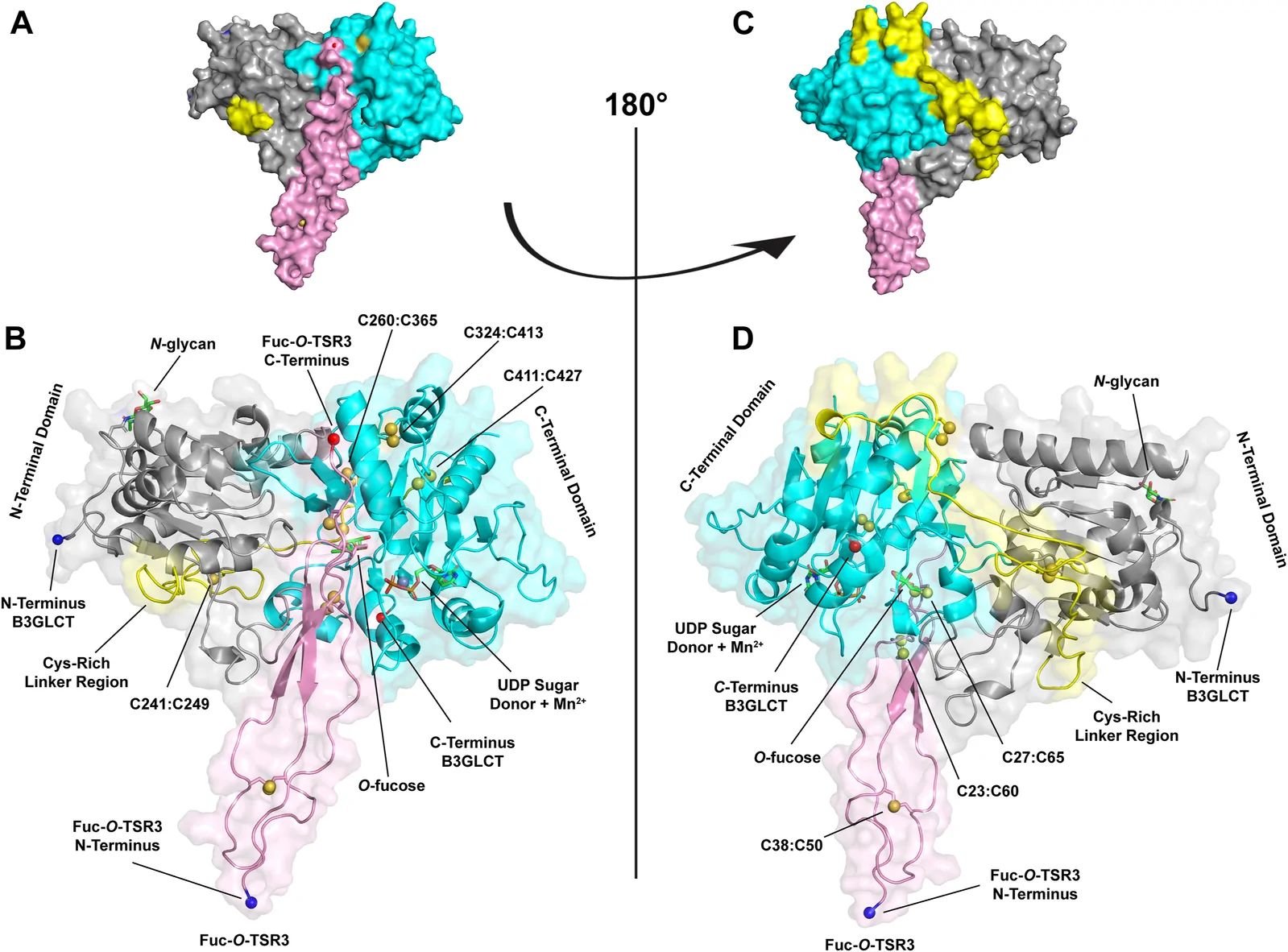
**The B3GLCT:UDP-Mn2^+^:Fuc-*O*-TSR3 complex.***A and C*, surface representation of the B3GLCT:UDP-Mn2^+^:Fuc-*O*-TSR3 complex with Fuc-*O*-TSR3 bound in the active-site cleft (*A*) and from behind (*C*). *Gray*: B3GLCT *N*-terminal domain, *Cyan*: B3GLCT C-terminal domain, *Yellow*: B3GLCT cysteine-rich linker region, *Pink*: Fuc-*O*-TSR3. *B*, cartoon representations of *panel A* with identical coloring. Mn^2+^ shown as *purple sphere*, UDP and fucose shown in stick representation. Cysteines represented by *yellow* spheres with disulfide bonds labeled. *D*, cartoon representation of *panel C*, colored and labeled identical to *panel B*.

The B3GLCT and Fuc-*O*-TSR3 proteins used for crystallography were highly pure, each exhibiting a single band on reducing gels (Fig. S1, *B* and *C*). In addition, unmodified TSR3 was modified by the addition of fucose to generate Fuc-*O*-TSR3 as an acceptor for B3GLCT (Fig. S1, *D* and *E*). Gel filtration and SEC-MALS analysis shows that monomeric B3GLCT forms a stable complex with Fuc-*O*-TSR3 (Figs. S2 and S3). The B3GLCT:UDP-Mn2^+^:Fuc-*O*-TSR3 complex was crystallized in the presence of UDP and Mn^2+^ and the structure was partially solved using molecular replacement with an AlphaFold (23) generated search model of B3GLCT. After placing one molecule of B3GLCT, a second round of molecular replacement using the crystal structure of Thrombospondin-1 Type 1 Repeat (PDB ID 3R6B) was used to complete the solution of the B3GLCT:UDP-Mn2+:Fuc-O-TSR3 complex, and the final structure was refined to a resolution of 2.06 Å (Table S2). The asymmetric unit of B3GLCT:UDP-Mn2^+^:Fuc-*O*-TSR3 contains a single chain of B3GLCT and Fuc-*O*-TSR3 (Fig. 2, *A*–*D*). The B3GLCT structure contains an N-terminal (residues 48–226) and C-terminal (residues 269–480) domain, both of which adopt a GT-A fold as previously proposed (13). The N- and C-terminal domains of B3GLCT are connected by a 42-residue linker region that wraps around the opposite face of the protein relative to the Fuc-*O*-TSR3 binding site (Fig. 2*C*). In B3GLCT, four disulfide bonds were identified: one within the linker region (C241:C249), one between the linker region to the C-terminal domain (C260:C365), and two within the C-terminal domain (C324:C413 and C411:C427) (Figs. 2*B*). Each GT-A domain is predicted to contain a single *N*-glycosylation site, but electron density was observed only for the *N*-acetyl-glucosamine of the glycan attached to Asn124 in the N-terminal domain. Unambiguous electron density was observed for a well-ordered UDP and a Mn^2+^ ion in the active site of the C-terminal domain, whereas the N-terminal domain lacked density for either a nucleotide or Mn^2+^.

The Fuc-*O*-TSR3 acceptor binds in a shallow groove formed between the N- and C-terminal GT-A domains of B3GLCT. The complex is reminiscent of the acceptor bound crystal structures of GT-B fold enzymes like POFUT1 and POFUT2, which bind their respective protein acceptors EGFs and TSRs in a similar shallow groove formed between the N- and C-terminal domains (Fig. S4) (24, 25, 26). The Fuc-*O*-TSR3 structure resembles previously determined TSR structures (Fig. S5, *A*–*I*). The TSR structure contains three disulfides formed by six cysteines (numbered C^1-6^) that are paired in the characteristic Group 1 TSR configuration C^1^:C^5^, C^2^:C^6^ and C^3^:C^4^, corresponding to C23:C60, C27:C65 and C38:C50 in Fuc-*O*-TSR3 (Figs. 2, *B*, *D* and S5, *A*–*E*) (27). The N-terminal residues are followed by the *O*-fucosylation consensus sequence located in a loop between Cys23 and Cys27, and two β-strands (β-strands B1 and B2) forming an anti-parallel β-sheet (Figs. 2, *B*, *D* and S5*A*).

### The active site is located in the C-terminal domain

**Figure 3:**
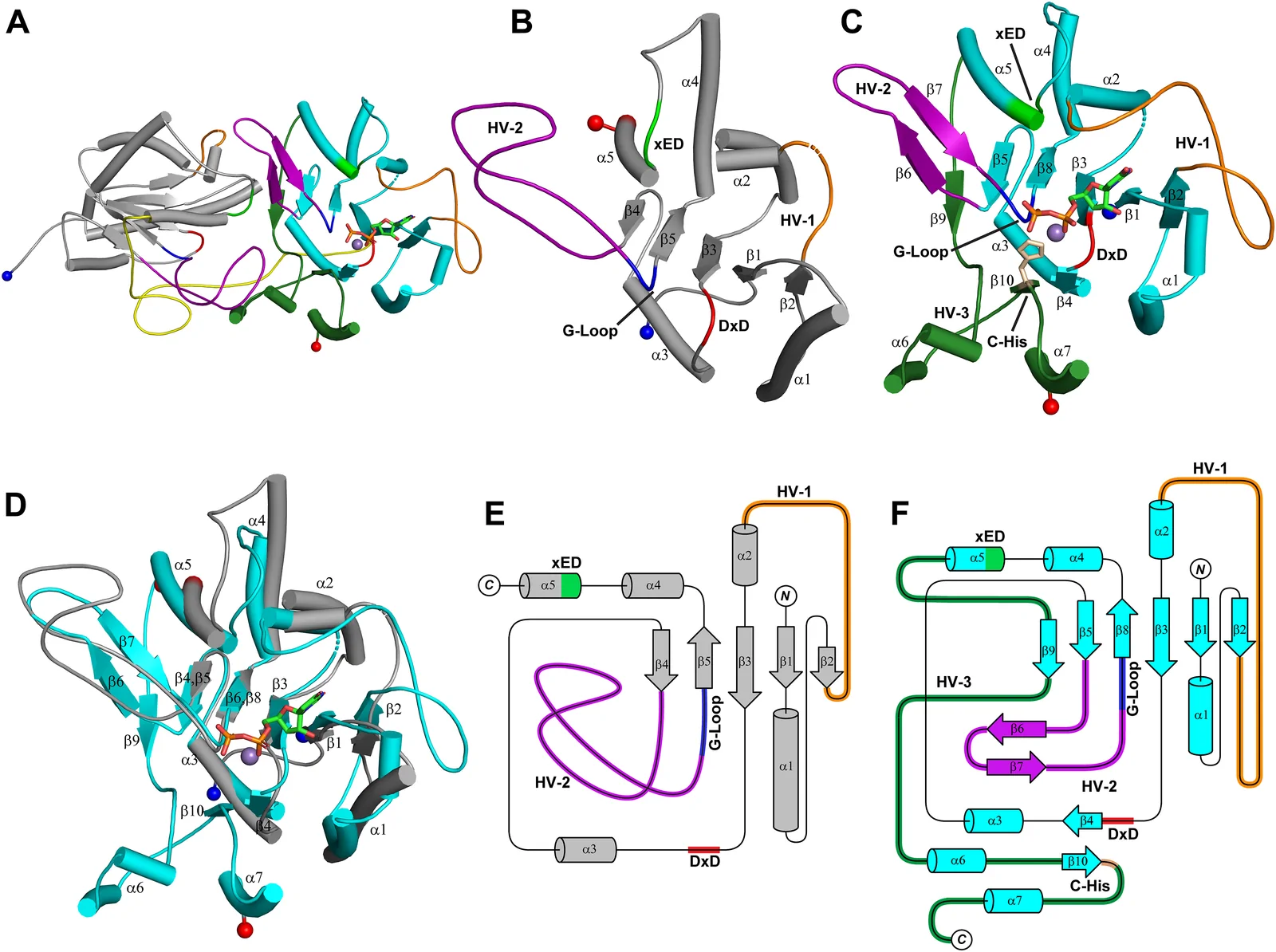
**Comparison of the B3GLCT N-terminal and C-terminal domain secondary structure.***A*, cartoon representation of the B3GLCT enzyme highlighting secondary structural elements and conserved GT-A domain elements. N-terminal domain in *gray*. C-terminal domain in *cyan*. Cysteine-rich linker region in *yellow*. Hypervariable Region-1 (HV-1) in *orange*, HV-2 in *purple*, and HV-3 in *dark green*. DxD motif in *red*, glycine-Rich loop (G-Loop) in *dark blue*, and xED motif in *light green*. C-terminal His (C-His) in tan. (*B* and *C*) Secondary structural elements and conserved GT-A domain elements of the N-terminal (*B*) and C-terminal (*C*) domains of B3GLCT. *D*, superimposition of the N- and C-terminal domains. *E*, two-dimensional map highlighting the N-terminal domain secondary structures and conserved GT-A domain elements. *F*, two-dimensional map highlighting the C-terminal domain secondary structures and conserving GT-A domain elements.

**Figure 4:**
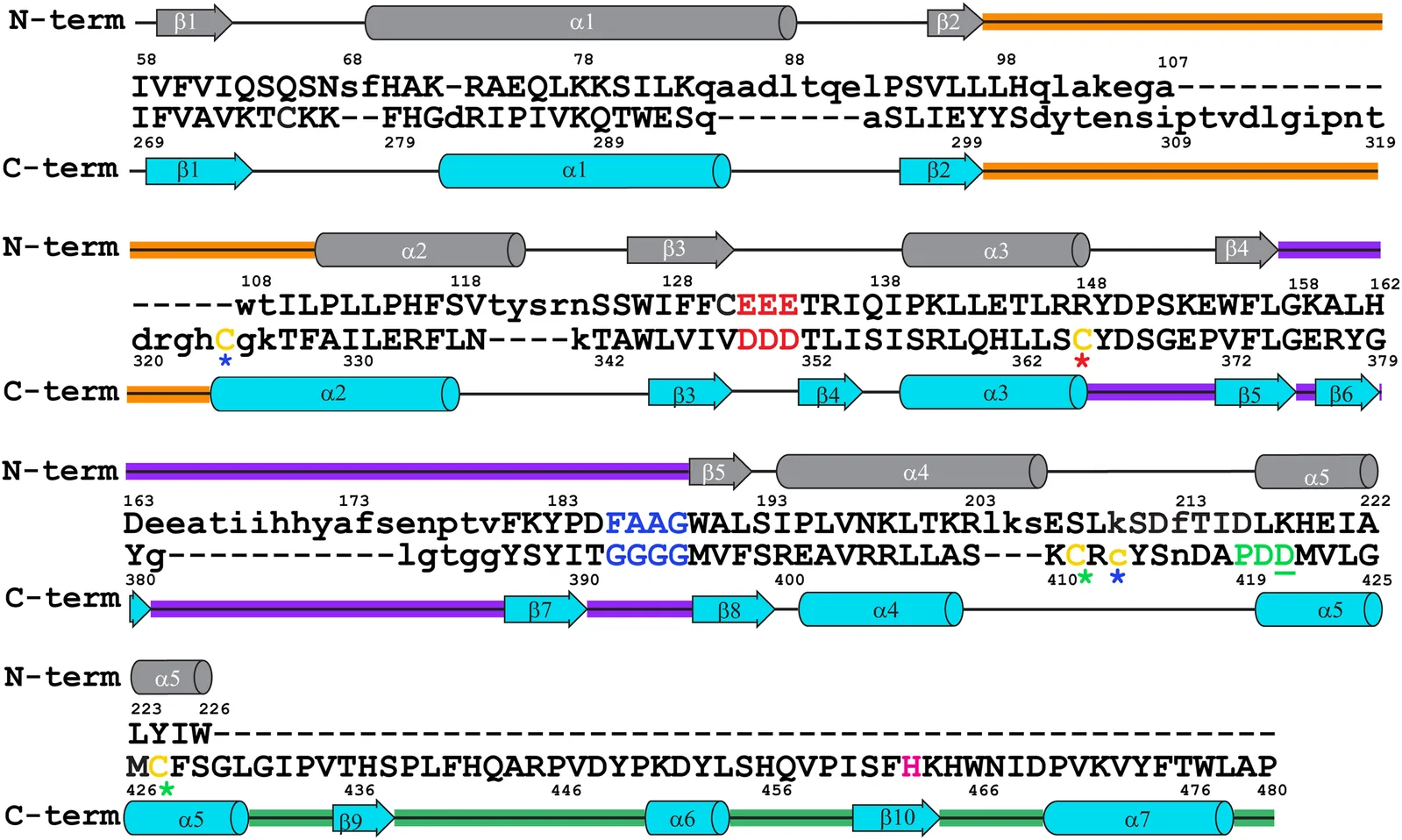
**Structure based sequence alignment of the N-terminal and C-terminal domains of B3GLCT.** The structure-based sequence alignment was performed using the TmAlign software (54). Uppercase letters identify structurally equivalent residues while lowercase letters identify structurally inequivalent residues. The N-terminal domain secondary structures (α-helices and β-strands) are colored in *gray*. The C-terminal domain secondary structures are colored in *cyan*. The HV-1, HV-2 and HV-3 regions are colored *orange*, *purple* and *dark green*, respectively. DxD motifs are shown as red letters in the sequence. The xED motif is colored *green*, and the catalytic base is underlined in *green*. Glycine-rich loops are depicted as *blue* letters and the C-terminal His is colored *pink*. Cysteines forming disulfide bonds are colored mustard *yellow*. *Blue*, *green* and *red* asterisks indicate the Cys324:Cys413, the Cys411:Cys427 and the Cys260:Cys365 disulfide bonds, respectively. Cys260 and the Cys241:Cys249 disulfide bonds are not shown since they are in the Linker Region.

**Figure 5:**
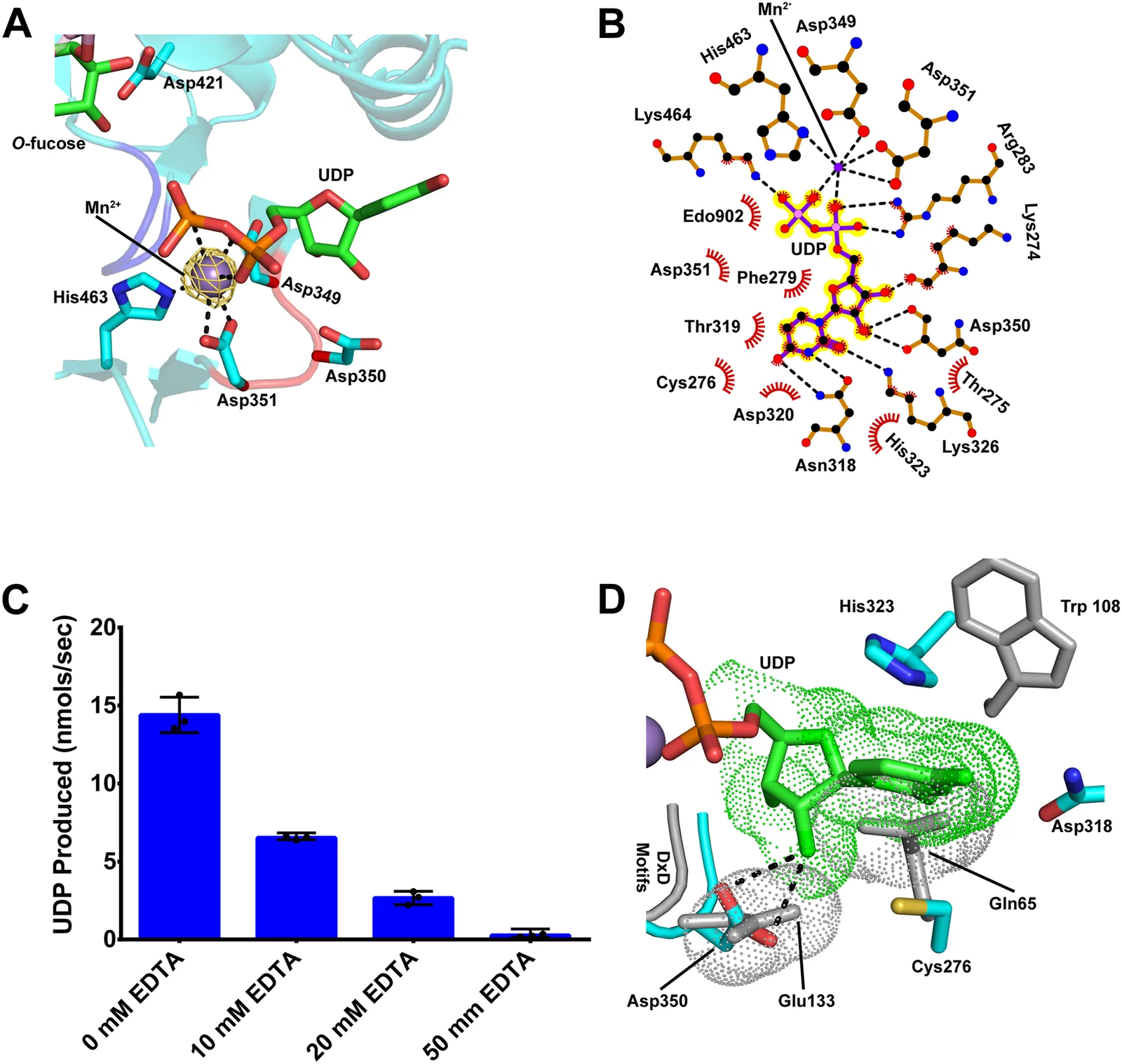
**Analysis of UDP, Mn^2+^ in the C-terminal domain active site.***A*, Mn^2+^ coordination in the active site of the C-terminal domain and a 2*F*_o_-*F*_c_ electron density map colored in *yellow* and contoured at the 5 σ level around Mn^2+^. Hydrogen bonds are indicated by *black dotted lines*. The DxD motif, the Glycine-rich loop and the Mn^2+^ are colored *red*, *dark blue* and *purple*, respectively. *B*, two-dimensional LigPlot (55) of B3GLCT residues interacting with UDP and Mn^2+^. UDP is highlighted in *yellow*. *C*, graph of activity of GFP-B3GLCT-WT using varying amounts of ethylene diamine tetra acetate (EDTA) for metal chelation. The high amounts of EDTA used to completely abolish B3GLCT activity suggests that the Mn^2+^ is tightly bound in the active site. Errors bars are reported as standard deviation of the mean and were calculated in GraphPad Prism v.6.01 (Siemens). *D*, steric clashes between UDP and residues from the N-terminal domain. The uridine and ribose rings of the UDP are shown as *green* van der Waals dots. The N-terminal domain residues are shown as *grey* van der Waals dots.

The C-terminal domain of B3GLCT conserves the essential GT-A motifs, including the Rossmann-like fold for UDP-sugar binding, the DxD motif and C-terminal His463 for coordinating Mn^2+^, the xED motif containing the catalytic base, the G-loop which contributes to the sugar binding in the active site, and the three hypervariable regions (HV) that are important for acceptor binding (Figs. 3, *A*, *C*, *F* and 4). The crystal structure of the B3GLCT:UDP-Mn2^+^:Fuc-*O*-TSR3 complex reveals electron density for the Mn^2+^ (Fig. 5*A*). The metal ion displays octahedral coordination geometry and interacts with three side chain oxygen atoms from the DxD motif (Asp349 and Asp351), the Nε2 from the C-terminal His463 and two oxygen atoms from the α- and β-phosphate groups of the UDP (Fig. 5, *A* and *B*). Although previous studies (13) suggested that B3GLCT activity was metal independent, the observed Mn^2+^ density prompted us to revisit this question. In support of the crystallographic evidence, metal chelation assays confirm that B3GLCT activity is metal dependent (Fig. 5*C*). The rest of the UDP makes extensive hydrogen bonds and packing interactions in the active site (Fig. 5*B*). Specifically, Asn318 in HV-1 forms two hydrogen bonds with the O4 hydroxyl and N3 nitrogen of the uracil ring (Fig. 5*B*). Lys326 hydrogen bonds with the O2 hydroxyl of the uracil portion of UDP. Asp350 (from the DxD motif) and the backbone carbonyl from Lys274 interact with the O2′ and O3′ hydroxyls of the ribose ring of UDP, respectively. Additionally, Arg283 and Lys464 form hydrogen bonds with the oxygens of the UDP diphosphate.

### The N-terminal domain likely evolved from the C-terminal domain and retains a vestigial active site

Consistent with a prior phylogenetic analysis (28) and despite low sequence identity (25.3%), the N- and C-terminal domains of B3GLCT are more similar to each other than to other GT31 CAZy family members. To further investigate the origin of the N-terminal domain, we performed comparative structural analyses by superimposing both domains from our crystal structure onto all other GT31 family structures. Superposition of the B3GLCT N- and C-terminal domains yielded an RMSD of 1.8 Å across 178 aligned Cα atoms (Fig. 3*D*). Superposition of the N-terminal domain with other GT31 structures resulted in an average RMSD of 2.7 Å, whereas the C-terminal domain showed a lower average RMSD of 1.7 Å against the same structures, and an RMSD of 0.98 Å when aligned with Manic Fringe (MFNG) (Fig. S6, *A*–*H*). These results suggest that the B3GLCT C-terminal domain most likely evolved from the three Fringe GTs which agrees with the closest phylogenetic ancestor based on previous findings (28). Furthermore, the relatively low RMSD between the two B3GLCT domains supports the hypothesis that the N-terminal domain evolved from the C-terminal domain from a possible gene duplication event and subsequently fused to the C-terminal domain, acquiring features that facilitate Fuc-O-TSR binding.

Despite the conservation of the GT-A fold, the N-terminal domain is lacking many features necessary for an active glycosyltransferase (Fig. 4). First, the aspartates in the DxD motif have diverged to glutamic acids (E132, E133, and E134), which are unable to form a coordination complex with a metal ion because of the additional carbon atom in the sidechain. In addition, the C-terminus of the fold is truncated after helix α5 and lacks the HV-3 loop which contains the His that contributes to metal binding in the C-terminal domain (Fig. 3, *B*, *D* and *E*) and other GT31 family enzymes (29, 30, 31) (Fig. S6, *B*–*F*). The lack of the metal cofactor would be sufficient to inactivate the enzyme, but there are additional substitutions Gln65 and Glu132 which would introduce steric clashes and prevent the binding of a nucleotide sugar (Fig. 5*D*). The N-terminal domain also misses the xED motif and the associated catalytic base, thus the absence of a nucleotide sugar binding site and catalytic residues means that the vestigial active site in the N-terminal domain is inactive (Fig. 4). The N-terminal domain is rotated ∼90° relative to the C-terminal domain to form a deep cleft between the domains for binding the TSR3 acceptor. The rotation of the N-terminal domain buries the vestigial active site in the interface between the two domains, where it forms a large, deep pocket that might contribute to domain flexibility in the complex (Fig. S7, *A*–*E*, Table S3).

### The Fuc-O-TSR3 binding site is formed by the HV loops from the N- and C-terminal GT-A folds

**Figure 6:**
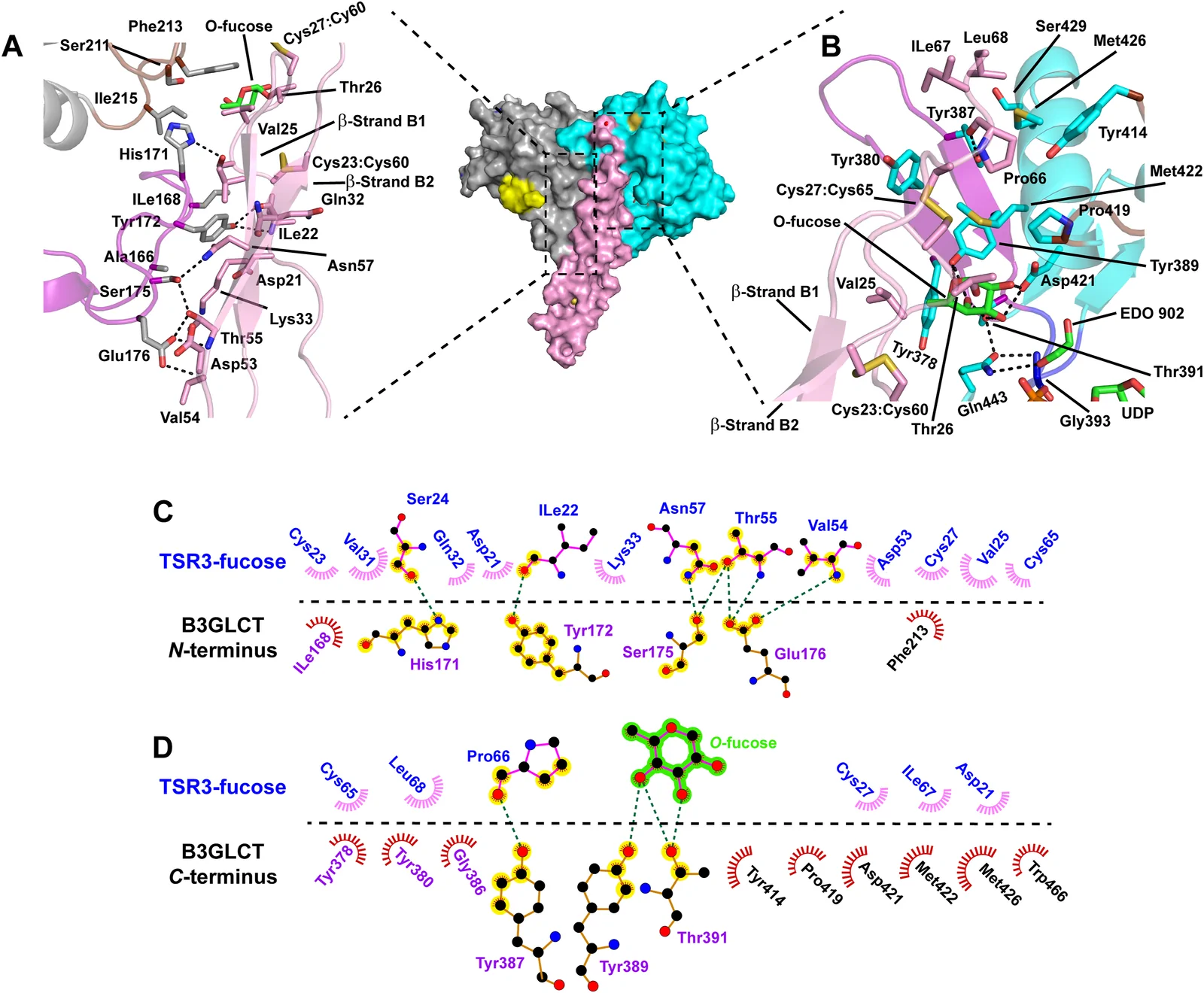
**The binding interface of B3GLCT and Fuc-*O*-TSR3.***A*, Cartoon depiction of the positive binding interactions between the N-terminal domain residues and Fuc-*O*-TSR3 residues. Fuc-*O*-TSR3 colored *pink*. HV-2 peptide backbone colored *purple*. Loop between α4 and α5 helices colored *brown*. *B*, Cartoon depiction of the positive binding interactions between the C-terminal domain residues and Fuc-*O*-TSR3 residues. For both (*A*) and (*B*) *black dotted lines* represent hydrogen bonds. *C*, Two-dimensional DimPlot (55) showing all interactions between Fuc-*O*-TSR3 (*top*) and the N-terminal domain (*bottom*). *D*, two-dimensional DimPlot showing all interactions between Fuc-*O*-TSR3 (*top*) and the C-terminal domain (*bottom*).

**Figure 7:**
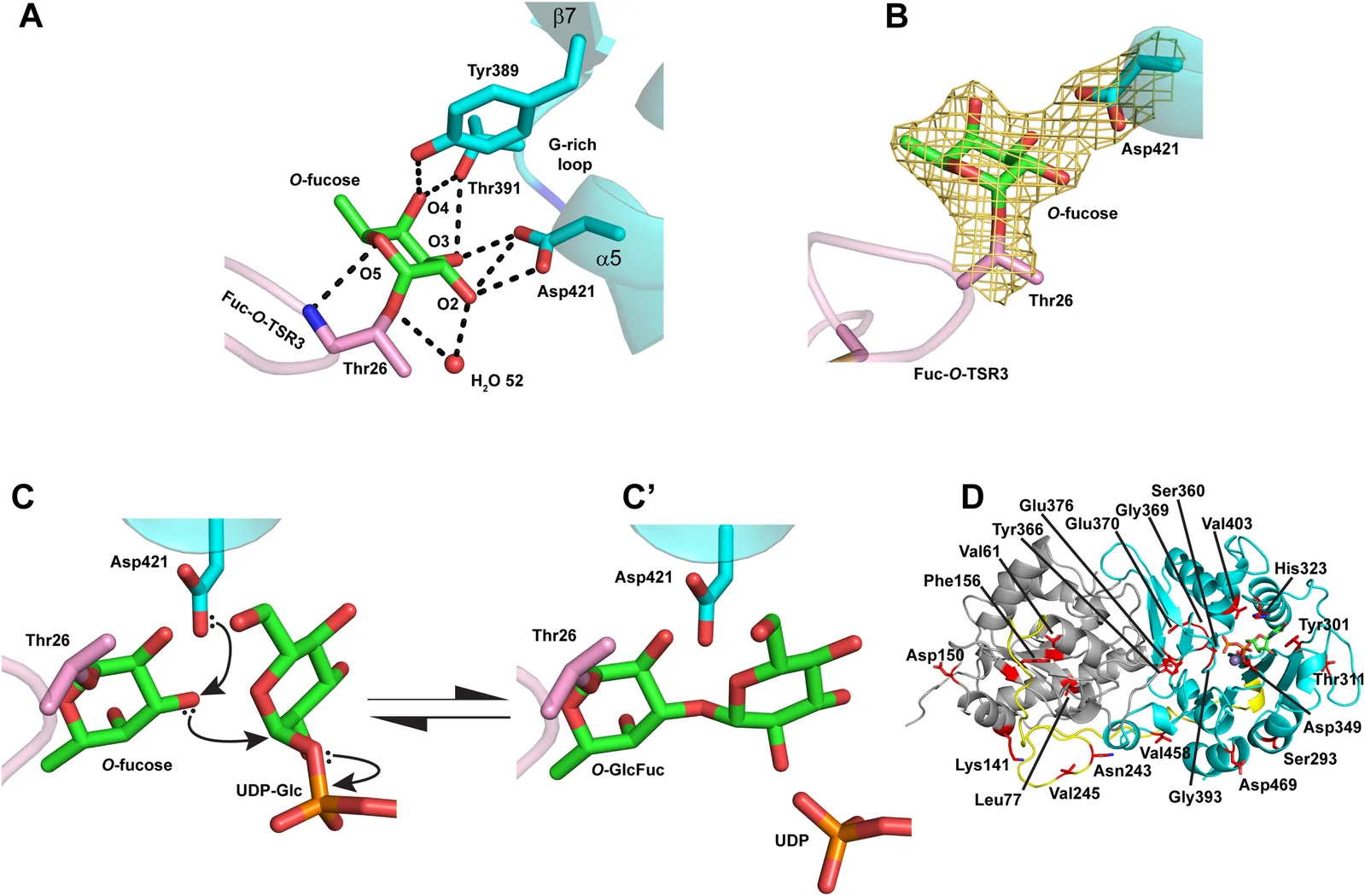
**Hydrogen bond analysis around *O*-fucose, a model for an inverting mechanism and prospective Peters Plus Syndrome variants.***A*, hydrogen-bonding network around *O*-fucose from B3GLCT residues in the active site. Hydrogen bonds are shown as *black dotted lines*. Water is indicated by a *red sphere*. *B*, 2*F*_o_-*F*_c_ electron density around the *O*-fucose and the catalytic base Asp421, contoured at the 1σ level and colored *yellow*. *C*, an AlphaFold3 predictive model for the inverting mechanism of B3GLCT activity upon the addition of glucose to Fuc-*O*-TSR3 and the product shown in (C′). *D*, *a* cartoon depiction of prospective Peters Plus Syndrome mutations shown in *red*.

The Fuc-*O*-TSR3 acceptor binding to the shallow groove buries 31 B3GLCT residues for a total of 871.2 Å^2^ of surface area (Table S4, *A* and *B*). Of these, 11 residues are from the N-terminal domain, and 20 residues are from the C-terminal domain. Most of the residues for binding the Fuc-*O*-TSR3 acceptor originate from the HV-2 regions of both domains (Fig. 6, *A* and *B*). This is consistent with previous work showing that the HV regions of GT-A fold enzymes have evolved for acceptor binding specificity (21, 22, 29). Figure 6 details all the Fuc-*O*-TSR3 interactions with the N- and C-terminal domain HV-2 residues. In addition, the N-terminal domain residues in helices α4, α5 and their connecting loop also contribute to Fuc-*O*-TSR3 binding (Fig. 6, *A* and *C*). From the C-terminal domain, both the HV-2 region and the α4-α5 loop contain residues that contribute to Fuc-*O*-TSR3 association (Figs. 7*A* and 6*B*, Table S4A). The O3 and O4 hydroxyls of the fucose moiety form hydrogen bonds with Tyr389 and Thr391 of the C-terminal HV-2 region loop (Figs. 7*A* and 6*B*, *D*). The O3 and O2 hydroxyls of the fucose form hydrogen bonds with Asp421. The backbone amide of Thr26 hydrogen bonds with the O5 oxygen and a water molecule forms hydrogen bonds with the O2 hydroxyl (Fig. 7*A*). In addition to the Fuc-*O*-TSR3 hydrogen bonding network observed in our structure, electron density was observed for the fucose moiety (Fig. 7*B*).

The contribution of the binding cleft residues was probed by substituting select N-terminal and C-terminal residues to alanine (Fig. S8, *A*–*C*). Selected residues for mutation were chosen by PISA analysis and residues from B3GLCT that were significantly buried upon Fuc-*O*-TSR3 binding (>50% buried) or formed hydrogen bonds with Fuc-*O*-TSR3 residues were mutated. As expected, several C-terminal domain mutants displayed significantly reduced B3GLCT activity (Fig. S9, *B* and *D*, Table 1). The most significant mutations involved Tyr389Ala and Gln443Ala, which nearly abolished enzymatic function. Importantly, the substrate saturation curve for Tyr389Ala displays non-saturatable kinetics against the acceptor, which means that the affinity for acceptor has been reduced to a point where we cannot saturate the enzyme to measure *k*_*cat*_ or *K*_*M*_ (Fig. S9, *B* and *E,* Table 1). This result also means that we cannot report the substrate (UDP-Glc) *k*_*cat*_ or *K*_*M*_ since we cannot satisfy the pseudo-first-order condition by saturating the enzyme with acceptor (Fig. S9, *D* and *F,* Table 1). The Tyr389 sidechain is essential since it directly forms hydrogen bonds with the fucose O4 and O5 hydroxyls, so the loss of this interaction is critical for acceptor binding. Similarly, Gln443 forms a hydrogen bond network with Thr391, the Gly393 backbone amide, and an ethylene glycol molecule from the cryoprotectant (EDO902) (Fig. 6*B*). EDO902 is positioned about where the glucose moiety from UDP-glucose would be bound, and disruption of these key bonds would be expected to impair glucose transfer to Fuc-*O*-TSR3. Thermal stability assays indicate Tyr389Ala also yields a less stable enzyme than the wild type, which is probably related to the loss of the packing interactions with the Tyr389 aromatic ring (Fig. S10). For the N-terminal domain mutants, S175A and I215A both slightly reduced *k*_*cat*_ and *K*_*M*_ against the acceptor (Fig. S9, *A* and *C*, Table 1). S175 and I215 both contribute hydrogen bonds or packing interactions to the Fuc-*O*-TSR3 residues, but these interactions are likely less direct as the Tyr389 and Gln443 interactions which likely explains their relatively small effect on activity.

**Table 1:** Kinetic Parameters for Wild-Type B3GLCT and Mutants using Fuc-*O*-TSR3 as acceptor substrate and UDP-Glucose as donor

Enzyme	GFP fluorescence^a^	Varying acceptor: Fuc-O-TSR3			Varying donor: UDP-glucose			
		k_cat_	K_0.5_	Rel. Cat. Activity^b^	k_cat_	K_M_	k_cat_/K_M_^d^	Rel. Cat. Activity^b^
WT	3269	62.4 ± 3.2	10.4 ± 0.93	N/A	66.8 ± 1.9	55.2 ± 3.9	1.21	N/A
***I168A***	1974	53.1 ± 13	6.74 ± 1.1	1.31	55.8 ± 5.9	312 ± 34	1.66	1.37
***H171A***	1971	56.5 ± 5.1	9.35 ± 0.70	1.01	61.6 ± 1.2	42.0 ± 4.0	1.47	1.21
***Y172A***	1008	55.9 ± 0.91	8.63 ± 0.28	1.08	68.0 ± 1.0	54.3 ± 4.1	1.25	1.04
***S175A***	2632	35.8 ± 1.1	5.06 ± 0.12	1.20	67.0 ± 3.0	143 ± 16.	0.467	0.386
***E176A***	3018	40.1 ± 0.93	5.61 ± 0.25	1.19	69.5 ± 0.28	130 ± 10	0.537	0.443
***S211A***	2914	43.6 ± 1.2	5.92 ± 0.20	1.22	69.2 ± 0.57	114 ± 12	0.607	0.501
***F213A***	2394	60.0 ± 9.1	10.5 ± 0.96	0.953	69.1 ± 4.0	55.5 ± 4.8	1.25	1.03
***I215A***	2221	27.2 ± 1.4	4.06 ± 0.17	1.11	80.3 ± 1.7	218 ± 15	0.369	0.305
Y380A	2686	53.1 ± 1.5	8.56 ± 0.51	1.03	63.6 ± 1.8	44.3 ± 3.5	1.43	1.18
Y387A	2332	53.7 ± 1.5	6.92 ± 0.44	1.30	65.0 ± 1.8	77.4 ± 10	0.840	0.693
Y389A	2134	N.D.^c^, ^d^	N.D.	N.D.	N.D.	N.D.	N.D.	N.D.
T391A	1865	18.9 ± 0.31	4.02 ± 0.09	0.781	43.0 ± 2.0	124	0.347	0.287
M422A	2761	46.4 ± 2.1	7.64 ± 0.32	1.01	50.0 ± 1.0	26.3 ± 2.9	1.91	1.57
M426A	3226	50.4 ± 1.5	6.64 ± 0.54	1.26	64.3 ± 1.1	51.4 ± 5.5	1.25	1.03
Q443A	3423	1.31 ± 0.03	1.25 ± 0.21	0.173	1.62 ± 0.06	3.40 ± 0.5	0.478	0.395
K464A	2842	17.2 ± 0.46	2.66 ± 0.08	1.08	19.3 ± 0.11	41.5 ± 3.7	0.464	0.383

Data from Varying Acceptor assays were fit to a substrate saturation curve. Data for Varying Donor assays were fit to a Michaelis-Menten curve. Both fits used non-linear regression in GraphPad Prism. N-terminal domain mutants are bold and italicized. C-terminal domain mutants are not bold and italicized.

a GFP fluorescence units are in milli-Absorbance Units (mAU).

b The equation for $relative\;catalytic\;efficiency=\left(\frac{{\left(\frac{k_{cat}}{K_{M}}\right)}_{mutant}}{{\left(\frac{k_{cat}}{K_{M}}\right)}_{wt}}\right)$.

c Mutant Y389A displayed nonsaturable kinetics against the acceptor. Therefore, the values for *k*_*cat*_ and *K*_*M*_ could not be detected (N.D.).

d For varying donor experiments, the *k*_cat_/*K*_M_ was determined with a subsaturating concentration of Fuc-*O*-TSR3 acceptor. Mutated residues that are bolded and italicized are from the N-terminal domain. The additional mutated residues are from the C-terminal domain All substrate saturation curves are technical triplicates. Errors bars for each substrate saturation curve are reported as standard deviation of the mean and were calculated GraphPad Prism v.6.01 (Siemens).

### B3GLCT catalysis and mechanism

Glycosyltransferases with GT-A folds catalyze either inverting or retaining reactions that define the anomeric configuration of the glycosidic product relative to sugar nucleotide donor. All GT31 structures published to date appear to function as metal-dependent inverting enzymes (22). To test whether B3GLCT also follows an inverting mechanism, we used AlphaFold 3 (AF3) (23) to model our B3GLCT:UDP-Mn2+:Fuc-O-TSR3 complex but employed UDP-Glc as donor rather than the donor analog in the crystal structure. AF3 superimposed the UDP-Glc onto the actual UDP in our crystal structure and positioned the glucose anomeric carbon 3.5 Å from the O3 hydroxyl of *O*-fucose on TSR3 and a distance of 2.5 Å from the O3 hydroxyl nucleophile of the Asp421 carboxyl oxygen. From this modeling, we hypothesize that B3GLCT catalysis proceeds *via* an inverting mechanism (Fig. 7, *C*–*C’*). Specifically, Asp421 acts as the catalytic base, deprotonating the O3 hydroxyl of *O*-fucose. The resulting oxyanion then attacks the glucose anomeric carbon of UDP-Glc, while electrons are displaced to the UDP pyrophosphate, leaving it with a net negative charge stabilized by interaction with the Mn^2+^ ion and completing the sugar transfer.

### Peters Plus Syndrome is caused by mutations in the B3GLCT gene

Peters Plus Syndrome is a CDG caused by mutations in the *B3GLCT* gene (14). Most of the mutations are splice site variants that produce truncated or unstable forms of the protein. A smaller subset consists of nonsense mutations that occur in conserved regions of B3GLCT. These include Asp349Gln, Asp349Gly, in the DxD motif, as well as Gly393Glu and Gly394Glu in the G-loop (Table S5). The two clinical mutations of Asp349 disrupt coordination of Mn^2+^ in the active site. The other two mutations in the G-loop introduce large, bulky side chains that sterically hinder UDP-glucose binding in the active site. In addition to these missense mutations, many other predicted missense variants have been suggested to be pathogenic on the ClinVar website (Table S5), and novel PPS mutations may be identified there in the future (Fig. 7*D*).

## Discussion

B3GLCT is an unusual GT-A fold enzyme because it is composed of two GT-A domains in close physical contact, forming a cleft between them that is used for acceptor substrate recognition. Of the roughly 220 known human glycosyltransferase genes, only nine contain two GT-A domains, and B3GLCT is the only one in which both domains belong to the GT-31 family (Table S1). What distinguishes B3GLCT from these other eight enzymes is that the GT-A domains in B3GLCT are joined by a linker region and the N- and C-terminal domains are physically adjoining one another, creating a cleft for binding Fuc-*O*-TSR substrates. The two GT-A domains from the other eight enzymes are not as intimately associated as the B3GLCT GT-A domains and some are considerably farther apart (Fig. S11, *A*–*I*). Furthermore, each domain of the other eight enzymes has a uniquely different substrate specificity, employing either different donor or acceptor substrates (20).

The structures of four of the eight other two-domain GT-A enzymes in humans have been determined to date. The structure of LARGE1, which adds repeating xylose and glucuronic acid units to α-dystroglycan, contains two GT-A domains that interact through an α-helix from each domain that are parallel to each other (Fig. S11*B*) (20). Its paralog, LARGE2, is predicted by AlphaFold 3 to adopt a similar fold (Fig. S11*C*). Beyond LARGE-1 and -2, the repeating chondroitin sulfate disaccharide units are synthesized by dimeric heterocomplexes formed between CHPF1 or CHPF2 and CHSY1 or CHSY3, and all four of these enzyme subunits also possess two GT-A domains. Notably, in each of these chondroitin sulfate synthase subunits, the GT-A domains are separated by a cystatin-like domain and a large α-helix, positioning the two domains far apart from one another (Fig. S11, *D*–*G*) (32, 33). Extending to other examples, COLGALT1 and COLGALT2, which transfer galactose to collagen, likewise contain two GT-A domains, and the structure of COLGALT1 has been solved; both this structure and the AlphaFold 3 prediction of COLGALT2 indicate that their GT-A domains interact *via* α-helices contributed by each domain, although the interface is less intimate than that observed between the B3GLCT domains (Fig. S11, *H* and *I*) (34). Thus, B3GLCT is atypical in that it possesses two GT-A domains, with the C-terminal domain most likely having diverged from the Fringe subfamily of GT31 enzymes. This C-terminal domain subsequently underwent gene duplication, giving rise to the N-terminal domain, followed by fusion into a tandem GT-A fold arrangement tethered by a linker region. These domains subsequently co-evolved—while retaining high similarity—to adopt distinct roles in substrate recognition and catalysis for a single Fuc-*O*-TSR acceptor substrate.

GT-B fold glycosyltransferases also contain two domains, both with Rossman-like folds. In GT-B fold enzymes, the N-terminal (A) domain has the predominant function of acceptor substrate recognition while the C-terminal (B) domain binds the sugar nucleotide donor. B3GLCT functions in a comparable fashion: the N-terminal domain primarily mediates TSR recognition and binding residues, while the C-terminal domain is responsible for both donor and additional TSR recognition as well as contributing the catalytic base for B3GLCT activity. In this way, B3GLCT achieves a functional arrangement reminiscent of GT-B fold glycosyltransferases but employs two GT-A folds instead. Importantly, the N-terminal domain of B3GLCT lacks the HV-3 region and the conserved C-His, though not all GT-A fold glycosyltransferases require C-His for divalent cation coordination and may substitute other alternative residues (21). Moreover, while both the N- and C-terminal domains in B3GLCT contain predicted xED motifs, only the xED in the C-terminal domain is appropriately positioned to serve as a catalytic base. Together, this suggests that the N-terminal domain has evolved from a canonical GT-A fold into a domain that more closely resembles the substrate-recognizing A domain of a GT-B fold enzymes, having lost the C-His motif and catalytic function associated with the xED motif.

Nearly 50 human proteins are known to contain TSRs, most of which harbor multiple tandem repeats. Almost half of these belong to the ADAMTS/TSL Superfamily of extracellular metalloproteases (35). A central role for many ADAMTS family members is turnover and degradation of the extracellular matrix (ECM), and dysregulation of their expression has been implicated in a range of diseases (36). Notably, loss of *O*-fucosylation on TSRs in many ADAMTS family proteins results in secretion defects to the ECM (6, 37, 38, 39, 40). Strikingly, the disease phenotypes caused by mutations in ADAMTS family members show considerable similarity and overlap with those observed in PTRPLS patients (41). Additionally, *O*-fucosylated TSRs have been confirmed in a handful of proteins from apicomplexan parasites in the Plasmodium and Toxoplasma genus’ that are involved in the transmission of malaria. (18, 42, 43). Due to their involvement of malaria transmission, the TSR domains of these proteins have specifically been studied as targets for antibody and vaccine design to combat and prevent malaria infections in humans (44, 45). These correlations to human health underscores the critical role of *O*-fucosylation in TSR function and highlights the significance in understanding how B3GLCT modifies TSRs.

## Experimental procedures

### Expression and purification of unlabeled and selenomethionine-labeled B3GLCT

A construct encoding GFP-B3GLCT (Uniprot Q6Y288, residues 33–467) was generated and inserted into the pGEn2-DEST expression vector by a strategy described previously (46). This construct encodes, from the N-terminal to C-terminal, a signal sequence in the N-terminus, an N-terminal 8xHis tag, AviTag, a “superfolder” GFP, a TEV protease recognition and cleavage site followed by B3GLCT (residues 33–467). This construct was transfected into GnT1-null HEK293F cells (ATCC# CRL-1573) in suspension. Cells were transfected in Freestyle 293 Expression Medium (Thermo Fisher; Cat. No. 12338018) supplemented with EX-CELL 293 Serum-Free Medium (Sigma-Aldrich Cat. No. 14571C) at a 9:1 (Freestyle:Excel) ratio. Briefly, 4 ug/ml of plasmid was mixed with the cells, followed 5 min later by 9ug/ml polyethylenimine (linear 25 kDa PEI, Polysciences, Inc, Warrington, PA) Transfected cells were incubated for 24 h at 37 °C, then culture volume was doubled with 9:1 (Freestyle:Excel) media supplemented with 4.4 mM valproic acid (2.2 mM final concentration). The cells were incubated at 37 °C and harvested 4 days post-transfection. Purification, GFP cleavage and *N*-glycan cleavage was performed as previously described (29).

### THBS1 Fuc-*O*-TSR3 expression and purification

A bacterial construct encoding human TSR3 from thrombospondin-1, pET20b^+^-hTSP1-TSR3, was previously designed (47). Briefly, TSR3 was expressed in chemically competent *E. coli* BL21 DE3 Star cells (Invitrogen; Cat. No. 44-0049) grown in LB Media at 37 °C. Cells were grown to an O.D._600_ of 0.6 and induced with 1 mM of Isopropyl β-D-1-thiogalactopyranoside (IPTG). Culture was incubated overnight at 20 °C. Cells were pelleted and resuspended in buffer consisting of 50 mM Tris-HCl pH 8.0, 1 mM phenylmethylsulfonyl fluoride (Thermo Fisher; Cat. No. 36978). After resuspension, cells were lysed on ice by sonication at 50% amplitude for 10 rounds of 10 s bursts with 2-min rest periods between each burst. Lysates were clarified by high-speed centrifugation, filtered through a 0.45 μM filter, then supplemented with NaCl to 0.5 M and Imidazole to 10 mM. Lysates were then purified and eluted from Ni-NTA beads with 250 mM imidazole in Tris-buffered saline. Elutions were further purified over a preparative HPLC equipped with a C18 reverse phase column (Agilent; Cat. No. 977250-102), flash frozen in liquid nitrogen and lyophilized. After lyophilization, TSR3 was resuspend in a buffer consisting of 50 mM Tris-HCl pH 7.5, 0.25 mM 2-mercaptoethanol (Fisher Scientific; Cat. No. BP176-100) and 0.2% sodium azide. TSR3 was modified *in vitro* by incubation with purified GFP-POFUT2 (46) and GDP-L-fucose disodium salt (Biosynth: Cat. No. MF01912) overnight at 37 °C in buffer consisting of 50 mM HEPES, 100 mM MnCl_2_.

### Crystallization and data collection

For crystallization, a truncated form of B3GLCT lacking the N-terminal signal peptide was fused with a superfolder GFP and used for protein expression (Fig. S1*B*). B3GLCT (10 mg/ml) in a storage buffer of 20 mM HEPES at pH 7.2, 100 mM NaCl was supplemented with 5 mM UDP, 5 mM MnCl_2_ and 0.23 mM Fuc-*O*-TSR3. The B3GLCT:UDP-Mn2^+^:Fuc-*O*-TSR3 complex was screened for crystallization conditions using the sitting drop vapor diffusion method with 2 μl drops containing a 1:1 protein:reservoir ratio. Crystals grew from a reservoir of 34% PEG 3350, 0.2 M dibasic ammonium phosphate and 0.5 M betaine. Crystals were transferred to the reservoir solution supplemented with 0.1 M sodium ascorbate and 6% cryoprotectant (1:1:1 ethylene glycol, DMSO and glycerol). The cryoprotected crystals were flash cooled in liquid nitrogen and data were collected at the SER-CAT 22-ID beamline at the Advanced Photon Source, Argonne, IL using an Eiger 16M detector and processed using XDS (48). Five percent of the data were set aside for cross-validation. The B3GLCT:UDP-Mn2^+^:Fuc-*O*-TSR3 complex crystallized in space group P6_3_22 and diffracted to 2.06 Å (Table S2).

### Structure solution and refinement

A model for B3GLCT was generated using the AlphaFold3 web server (23). The resulting solution was trimmed of loop regions at the N- and C-termini and used as the search model for B3GLCT in the B3GLCT:UDP-Mn2^+^:Fuc-*O*-TSR3 complex by using the molecular replacement program, Phaser (49). The B3GLCT molecular replacement solution was subsequently used as a partial solution to place the Fuc-*O*-TSR3 moiety in B3GLCT:UDP-Mn2^+^:Fuc-*O*-TSR3, also using Phaser (49). The available structure of Thrombospondin-1 Type 1 Repeats (PDB ID 3R6B) (50) was used as the search model for Fuc-*O*-TSR3. The resulting model for B3GLCT:UDP-Mn2^+^:Fuc-*O*-TSR3 was subjected to rigid body refinement using Phenix (49), followed by several rounds of automated structure refinement and iterative manual fitting using Coot (51). The NH_2_-terminus (residues 1–47) and C-terminus (residues 481–498) of the protein in B3GLCT:UDP-Mn2^+^:Fuc-*O*-TSR3 were disordered and left unmodeled. Loop regions containing residue 104 and residues 337 to 340 were unresolved in the electron density and also left unmodeled. The *F*_*o*_*-F*_*c*_ difference density map was used to place the UDP and Mn^2+^ ion bound to B3GLCT. An *omit* map calculated after a simulated annealing procedure confirmed that residue Lys464 in B3GLCT was a true Ramachandran outlier. In the Fuc-*O*-TSR3 model, amino acid residues 8 and 68 originate from the expression construct while residues 9 to 67 correspond to THBS1 residues 490 to 548. The final model for B3GLCT:UDP-Mn2^+^:Fuc-*O*-TSR3 (Table S2) was obtained by refining the B-factors using TLS (52).

### SEC-MALS experiments

Size Exclusion-Multiple Angle Light Scattering (SEC-MALS) was used to analyze the oligomeric sizes of the WT GFP-B3GLCT. B3GLCT WT was expressed in WT HEK293F suspension cells as described above except GFP and N-glycans were not cleaved. Clarified, conditioned media was subject to Ni-NTA batch binding, and B3GLCT was eluted with Tris-HCl pH 8.0, 250 mM Imidazole, 50 mM NaCl. Protein was further purified by size exclusion chromatography (SEC) using an AKTA Pure 25 FPLC (General Electric; Cat. No. 29018224) equipped with a HiLoad 16/600 Superdex 200 pg SEC column in 1X TBS pH 8.0. Glycerol was added to a final concentration of 20%, enzyme aliquoted, and frozen at −80 °C. The protein was concentrated to 1 mg/ml in a buffer consisting of 1xTBS, pH 7.5. GFP-B3GLCT was loaded onto a Cytiva Superdex 200 column (Cytiva, Cat. No. 28990944) and run with a buffer containing 25 mM HEPES pH 7.5 and 150 mM NaCl, at a flow rate of 0.2 ml/min. In-line light scattering was measured using a MiniDAWN TREOS detector (Wyatt Technologies) and differential refractive index using a Optilab rEx detector (Wyatt Technologies). Data analysis was performed using the ASTRA software package 6.0 (Wyatt Technologies).

### B3GLCT enzyme assays

In total, 16 mutant constructs were generated with single Ala point mutations (8 in the N-terminal domain and 8 in the C-terminal domain) by Genscript laboratories. B3GLCT WT control and each mutant were expressed and purified in WT HEK293F suspension cells as described above for SEC-MALS experiments except for concentrating to 1 mg/ml. Kinetic experiments were performed using UDP Glo Assay kits (Promega; Cat. No. V6992) with 600 nM of B3GLCT and varying or saturating concentrations of donor and acceptor substrates as appropriate. Assays were performed in 50 mM Tris pH 8.0. To determine the *K*_M_ and V_max_ for UDP-glc, a sub-saturating concentration of 30 μM Fuc-*O*-TSR3 acceptor substrate had to be used due to limited supply of this reagent. UDP-Glc donor concentrations were varied between 0.9 μM and 1000 μM. To determine the *K*_M_ and V_max_ for Fuc-*O*-TSR3, a saturating concentration of 200 μM UDP-Glc was used with concentrations of Fuc-*O*-TSR3 ranging from 0.23 μM to 30 μM. EDTA inhibition assays were performed in the presence of a saturating 200 μM UDP-Glc and 30 μM Fuc-*O*-TSR3 with varying concentrations of EDTA, incubated overnight at 4 °C. Data from assays were fit either to a Michaelis-Menten curve or a substrate saturation curve as appropriate using non-linear regression in GraphPad Prism v.6.01 (Siemens).

### Protein stability assays

For protein stability assays, B3GLCT WT and mutants were expressed and purified as described above in B3GLCT enzyme assays. All reactions were prepared using 0.2 mg/ml of enzyme mixed with 5X SYPRO Orange ThermoFluor (Thermo Fisher) in 1X TBS using a reaction size of 25 μl. A temperature range from 10 to 95 °C that increased 0.5 °C/s was employed on a Bio-Rad CFX96 real-time qPCR machine. The graph in Fig. S12 was generated by and exported out of CFX Maestro software (Bio-Rad).

## Data availability

All data are included in the manuscript and Supporting Information.

## Supporting information

This article contains supporting information (8, 12, 13, 14, 20, 24, 29, 31, 33, 34, 53, 56, 57, 58, 59, 60, 61, 62, 63).

## Conflict of interest

The authors declare that they have no conflicts of interest with the contents of this article.
