# Supplementary material for: Crystal structure of a β1,3-Glucosyltransferase reveals an unusual substrate recognition by a two-domain GT-A fold glycosyltransferase
Source: J Biol Chem. 2026 Aug 10;302(10):113418. doi: 10.1016/j.jbc.2026.113418 (PMC13597200; doi:10.1016/j.jbc.2026.113418)
Supplement: Supplementary file 1 [file mmc1.pdf]

Supporting Information for:

Crystal structure of a β1,3-Glucosyltransferase reveals an unusual substrate recognition by a two-domain GT-A fold glycosyltransferase

Steven J. Berardinelli, Renuka Kadirvelraj, Kelvin B. Luther, Zhongwei Gao, Digantkumar Chapla, Chin Huang, Daniel M. Tehrani, Ao Zhang, Kelley W. Moremen, Zachary A. Wood, Robert S. Haltiwanger

Contents:

SI Materials and Methods

Tables: S1-S5

Supplemental Figures S1-S11

Table S1: List of all human glycosyltransferases with two GT-A domains, or a GT-A and GT-B domain.

Protein name	GT families	GenBank ID	Uniprot ID
**Human GTs with 2 GT-A domains**			
Beta 1,3-glucoslytransferase (Beta3Glc-T) (EC 2.4.1.-) (Beta-3glucosyltransferase)	GT31, GT31	AAO37647.1	Q6Y288
Chondroitin sulfate synthase 1 (ChSy-1;CSS1;CHSY1;CHSY;KIAA0990)	GT31, GT7	AAQ88893.1	Q86X52
Chondroitin sulfate synthase 3 (CSS3;ChSy-2;CHSY2)	GT31, GT7	BAC98832.1	Q70JA7
Chondroitin sulfate synthase 2 (CSS2;ChPF;CHPF;FLJ22678)	GT31, GT7	AAH21223.2	Q8IZ52
Chondroitin sulfate glucuronyltransferase (CHPF2;CSGLCAT;KIAA1402)	GT31, GT7	AK056331.1	Q9P2E5
Procollagen galactosyltransferase 2 (EC 2.4.1.50) (Collagen beta(1-O)galactosyltransferase 2) (ColGalT 2)	GT2, GT25	AAG60609.1	Q8IYK4
Procollagen galactosyltransferase 1 (EC 2.4.1.50) (Collagen beta(1-O)galactosyltransferase 1) (ColGalT 1)	GT2, GT25	NP_078932.2	Q8NBJ5
Bifunctional a-1,3-xylosyltransferase / B-1,3-glucuronylltransferase (LARGE1; Large1)	GT8, GT49	CAA07571.1	O95461
Bifunctional a-1,3-xylosyltransferase / B-1,3-glucuronylltransferase (LARGE2)	GT8, GT49	BAC04675.1	Q8N3Y3
**Human GTs with a GT-A and GT-B domains**			
UDP-GlcUA:B-glucuronyltransferase / UDP-GlcNAc: a-N-acetylglucosamynaltransferase (exostosin-2; Ext2)	GT47, GT64	AAB07008.1	Q93063
UDP-GlcUA:B-glucuronyltransferase / UDP-GlcNAc: a-N-acetylglucosamynaltransferase (exostosin-1; Ext1)	GT47, GT64	AAB62283.1	Q16394
Exostosin-like 3 (Extl3;Extr1)	GT47, GT64	AAC39598.1	O43909
Exostosin-like 1 (Extl1)	GT47, GT64	AAC51141.1	Q92935

Table S2: Data collection and refinement statistics for B3GLCT:UDP-Mn2⁺:Fuc-*O*-TSR3

**Data collection**	**B3GLCT:UDP-Mn2⁺:Fuc-*O*-TSR3**
Wavelength (Å)	1.000
Space group	P 6_3 2 2
Unit cell dimensions (a, b, c in Å and angles in °)	133.9,133.9,137.8 and 90, 90, 120
Completeness (%)	99.8 (99.3)[a]
Total number of reflections	831342 (136039)
Unique reflections	45560 (7200)
Redundancy	18.2 (18.9)
I / σ(I)	21.1 (1.0)
R_{meas}[b] (%)	8.9 (337.4)
$CC_{1/2}$ (%)	99.9 (54.8)
**Refinement**	
Resolution (Å)	2.06
R_{work} / R_{free}	0.199 / 0.226
No. of atoms Protein / Ligand / Water	3904 / 60 / 112
B-factors (Å^2) Protein / Ligand / Water	65.5 / 63.6 / 60.7
**Stereochemical Ideality**	
Bond lengths (Å^2)	0.01
Bond angles (°)	0.93
Ramachandran favored (%)	97.71
ϕ,ψ Additionally allowed (%)	2.08
ϕ,ψ Outliers (%)	0.21
PDB Accession code	12EM

[a]Values in parentheses are for the highest-resolution shell (2.18 – 2.06 Å).

[b]R_{meas} is the redundancy independent merging *R*-factor of Diederichs and Karplus (1).

Table S3: List of all residues forming the deep pocket between N- and C-terminal domains

N-terminal domain			Cysteine rich linker region			C-terminal domain		
**Residue No.**	**Side Chain**	**Main Chain**	**Residue No.**	**Side Chain**	**Main Chain**	**Residue No.**	**Side Chain**	**Main Chain**
Gln63	y	y	Phe253	y	n	Leu361	y	n
Gln65	y	n	Ser255	y	n	Tyr366	y	n
Ser64	n	y	Phe256	n	y	Glu376	y	n
Ser66	y	y	Pro258	y	n	Arg377	y	n
Asn67	y	n				Try378	y	y
Phe69	y	n				Ser388	y	y
His70	y	n				Gly425	y	n
Arg73	y	n				Phe428	y	y
Leu98	y	n				Ser429	y	y
His99	y	n				Ile433	n	y
Ala102	y	n				Pro434	y	n
Glu105	y	n				Val435	y	y
Ala107	y	n				His437	y	y
Trp108	y	y				Ser438	y	n
Glu132	y	n				Pro439	y	y
Glu133	y	y				Leu440	y	n
Glu134	y	y				Phe441	n	y
Ile169	y	n				His442	y	n
Asp185	y	n				Tyr449	y	n
Phe186	y	n				Tyr453	y	n
Ala187	y	n				His456	y	y
Thr214	y	y				Gln457	y	n
Ile215	y	y						
Asp216	y	n						
Leu217	y	y						
Lys218	y	y						

Table S3: Residues from each domain that contribute to the outer surface of the deep pocket between the N- and C-terminal domains of B3GLCT. y=yes, atoms from the side chain or main chain contribute to the outer surface area of the cavity; n= no, atoms from the side chain or main chain do not contribute to the outer surface area of the cavity for that residue.

Table S4A: PISA results for B3GLCT binding interface

B3GLCT Interface Residues	Interface Area (Å²)	$\Delta^i G$ (*Kcal/mol*)	$\Delta^i G$ (*P-value*)	Hydrogen bonds & salt bridges	Polar residues in interface	Non-polar residues in interface
31	871.2	-453.0	0.072	9	***E165***, ***H171***, ***S175***, ***E176***, ***K210***, ***S211***, R321, T384, D421, S429, R445, K464, N467	***A166***, ***I168***, ***Y172***, ***P213***, ***I215***, Y380, G381, G385, G386, Y387, Y389, Y414, P419, M422, V423, M426, G430, W466

Table S4B: PISA results for Fuc-*O*-TSR3 binding interface

Fuc-*O*-TSR3 Interface Residues	Interface Area (Å²)	$\Delta^i G$ (*Kcal/mol*)	$\Delta^i G$ (*P-value*)	Hydrogen bonds & salt bridges	Polar residues in interface	Non-polar residues in interface
26	867.6	-33.4	0.155	9	D21, S24, T26, Q32, K33, D53, T55, N57, N61, Q63	P19, W20, I22, C23, V25, C27, G28, G29, G30, V31, G52, V54, C65, P66, I67, L68

Table S5: List of PTRPLS point mutations and predicted ClinVar mutations in B3GLCT and their predicted effects

N-terminal domain				Linker Region				C-terminal domain			
**Residue No.**	**AA**	**Mutation**	**Effect**	**Residue No.**	**AA**	**Mutation**	**Effect**	**Residue No.**	**AA**	**Mutation**	**Effect**
61	V	I	f	232	P	A	d	286	I	V	p
112	P	L	h	235	T	I	b	301	Y	F	b
115	P	L	h	239	E	G	b,d	311	T	I	b,d
141	K	E	b	243	N	S	b,d	323	H	R	s
150	D	E	b,d	245	V	M	p	337	R	H	u
156	F	I	c	255	S	Y	c,s	343	A	S	s
168	I	M	f,s					**349**	**D**	**G**	a
								**349**	**D**	**N**	a
								**366**	**Y**	*****	t
								368	S	A	?
								369	G	S	r
								376	E	G	b,c
								380	Y	*	t
								389	Y	*	t
								**393**	**G**	**E**	r
								**394**	**G**	**E**	r
								403	V	I	p,s
								440	L	V	c,s
								457	Q	R	f,s
								458	V	I	s
								469	D	Y	h
								483	E	K	u

Table S5: Residues that are in bold are previously known point mutations (2-4). In the effects column are possible structural effects that may occur due to the predicted mutation. a=ablates catalytic activity; b=breaking of hydrogen bond(s); c=creates or enlarges an endogenous cavity; d=creates disorder in a loop segment; f=fills endogenous cavity; h=disruption of helix capping; p=prevents hydrophobic packing; r=creates more rigidity in a loop segment; s=steric hindrance or clashes; t=truncating mutation; u=unmodeled in our crystal structure. The underlined D349G and G393E have been shown to be enzymatically dead in vitro (4).

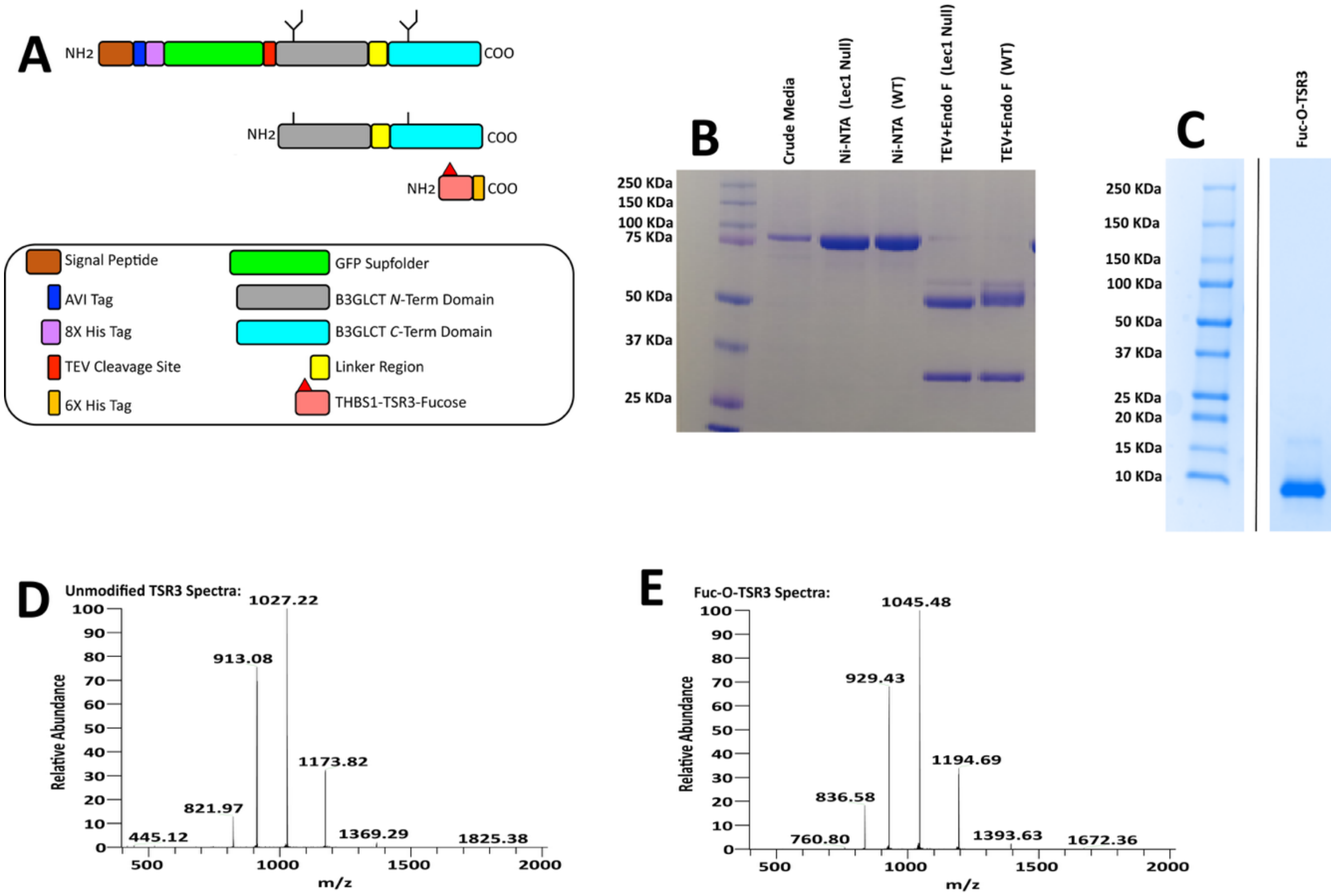

Figure S1: Description of proteins used for crystallography. (A) Domain maps of the GFP-B3GLCT, GFP-cleaved B3GLCT and THSB1 Fuc-*O*-TSR3. GFP-cleaved B3GLCT and THSB1 Fuc-*O*-TSR3 were used for crystallography. GFP was removed by TEV cleavage. *N*-glycans were trimmed by cleavage with Endo F. THSB1 TSR3 was bacterially expressed then modified in vitro with fucose (red triangle). (B) Representative gel showing crude expression, Ni-NTA purification and cleavage products of GFP-B3GLCT from wild type and Lec1 null 293F cells. B3GLCT from Lec1 null cells were used for crystallization due to better Endo F cleavage of *N*-glycans (Compare lanes 5 and 6). (C) Coomassie stained gel of Fuc-*O*-TSR3 after fucose modification and purification. (D) Spectra of TSR3 before TSR3 fucose modification (E) Spectra of TSR3 after fucose modification shows near-complete modification with fucose by native mass spectrometry.

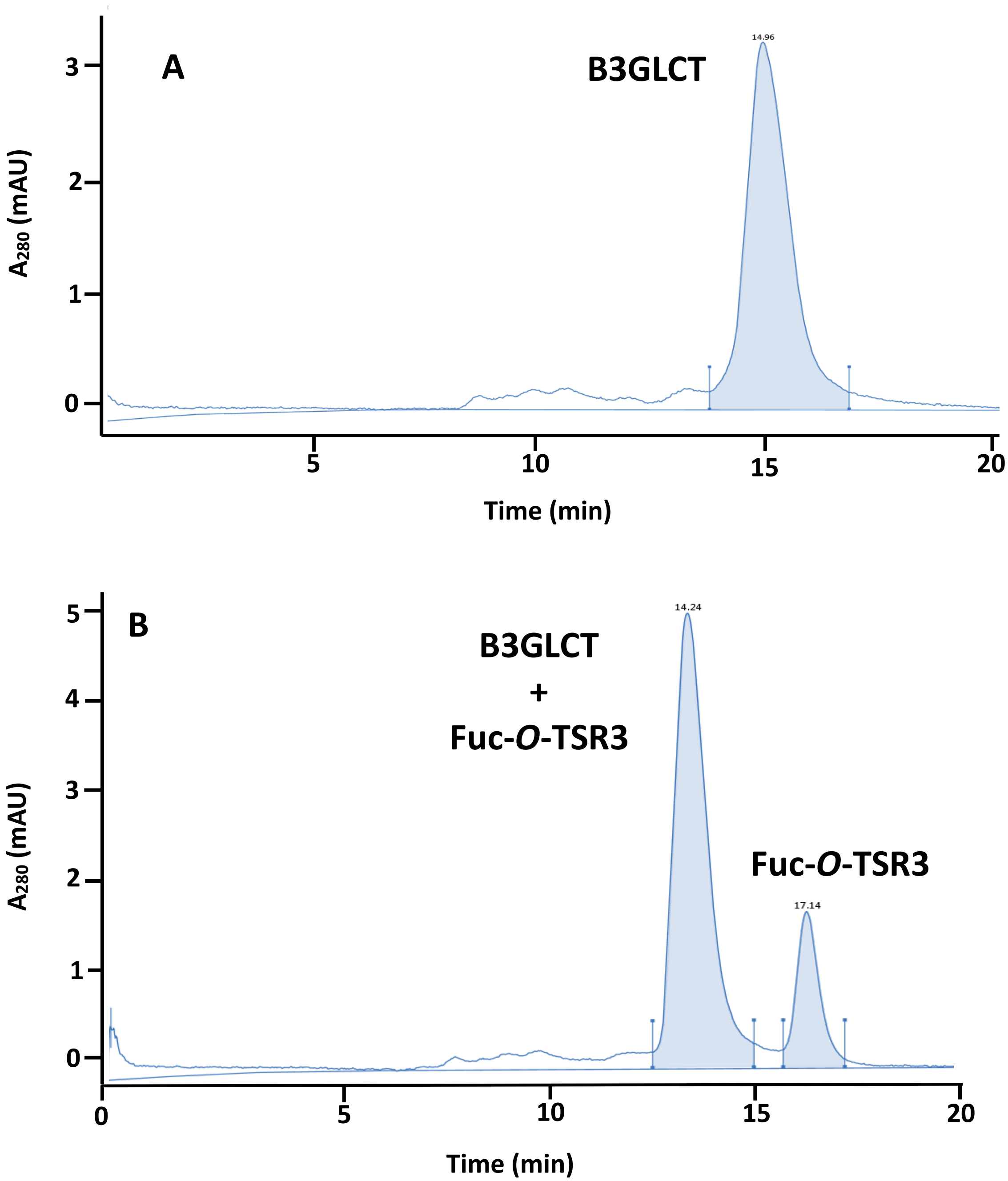

Figure S2: Gel Filtration experiments showing B3GLCT and TSR3-fucose binding. (A) Size exclusion chromatography resulted in B3GLCT having a retention time of roughly 15 min. (B) When incubating B3GLCT and Fuc-*O*-TSR3, a complex formed resulting in a retention time of roughly 14 min. An additional peak at roughly 17 minutes corresponds to Fuc-*O*-TSR3.

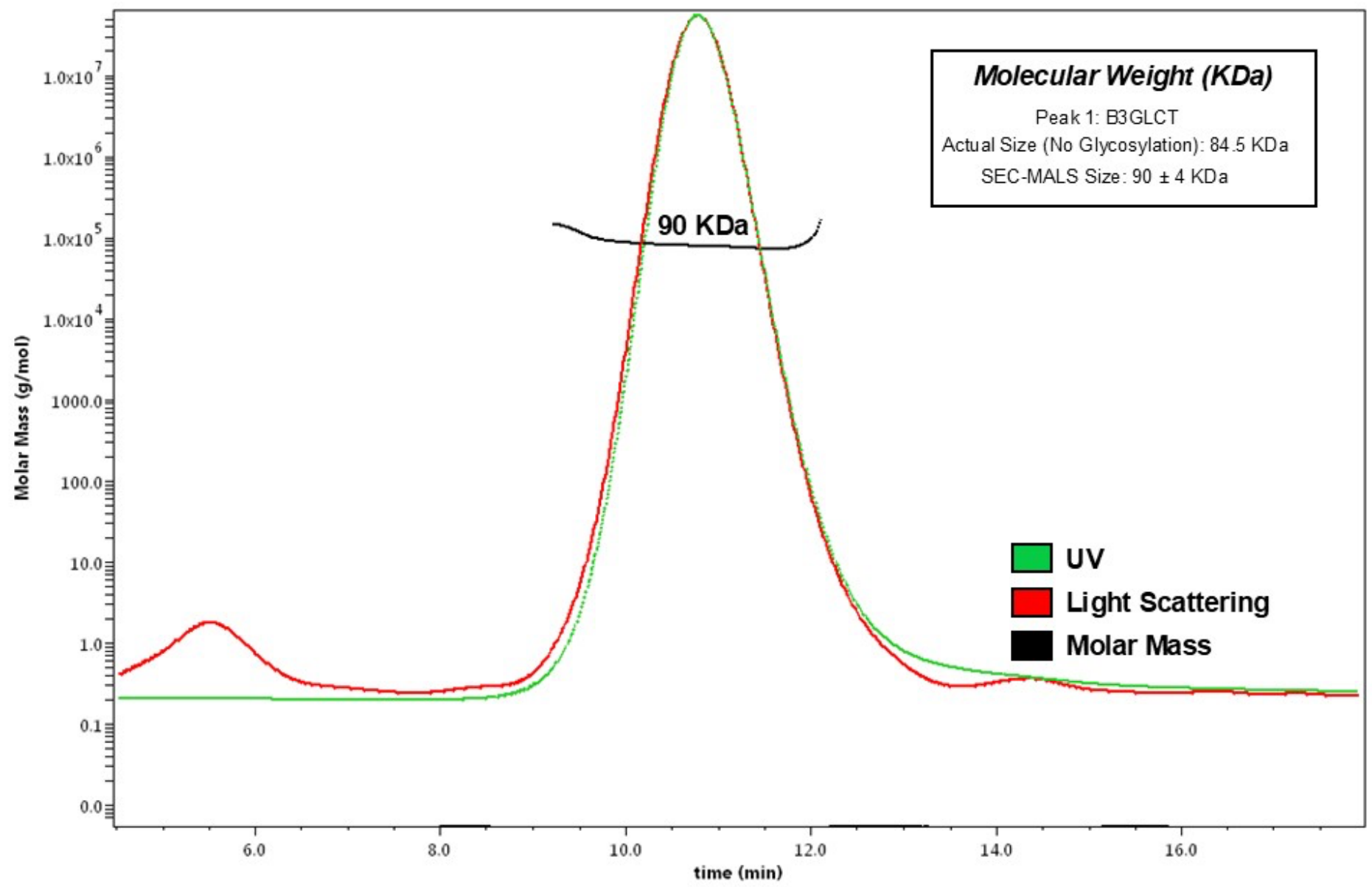

Figure S3: SEC-MALS reveals B3GLCT forms a monomer. (A) GFP-B3GLCT eluted at roughly 10.8 minutes which corresponds to a 90 ± 4 KDa monomer indicating that GFP-B3GLCT does not form a higher order structure (dimer, trimer etc.) with itself.

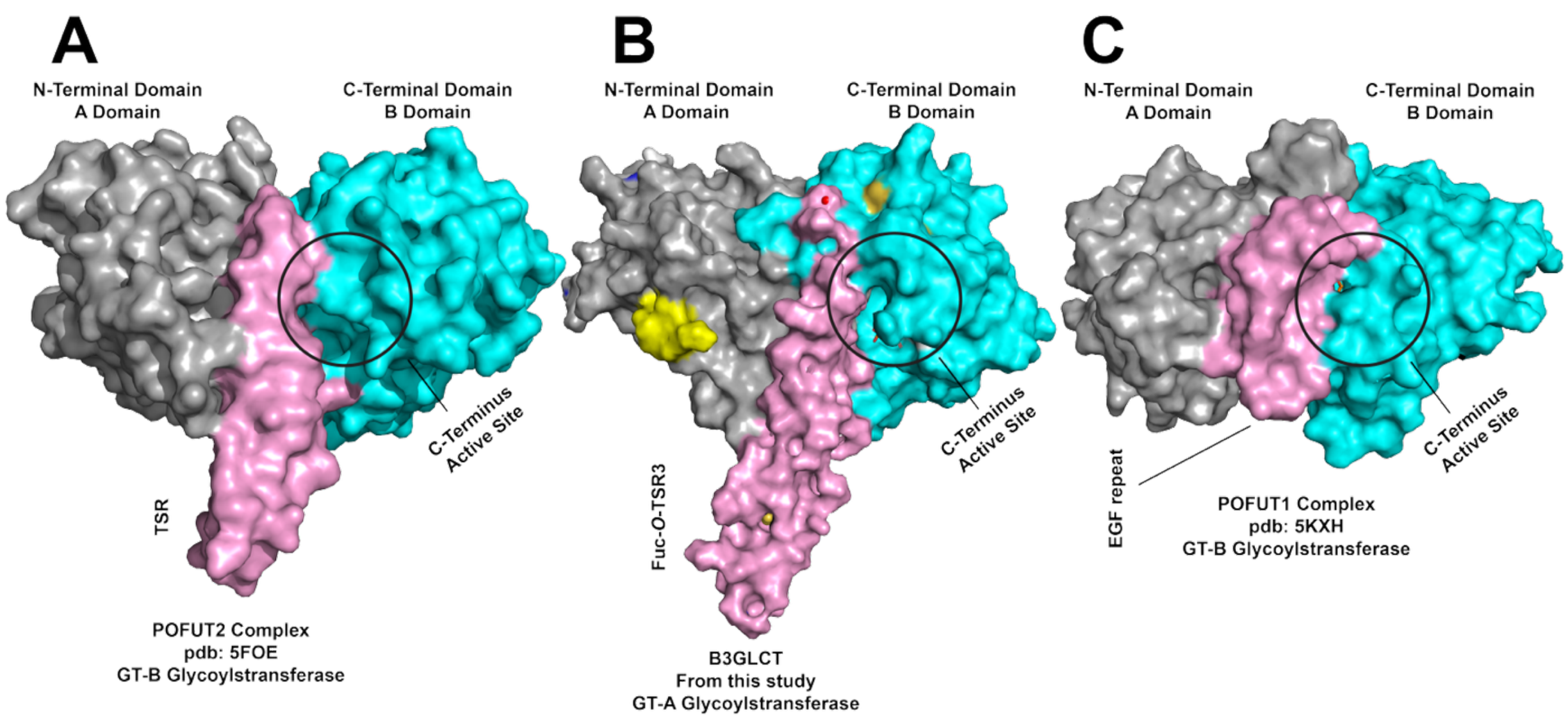

Figure S4: B3GLCT is similar in domain structure to POFUT1 and POFUT2. (A) The crystal structure of *C. elegans* POFUT2 and a TSR complex (5). (B) The crystal structure of the B3GLCT:UDP-Mn2^{+}:Fuc-*O*-TSR3 complex from this study. (C) The crystal structure of POUFT1 and an EGF complex (6). Both POFUT1 and POFUT2 are GT-B fold domains containing an A domain and a B domain. The A and B domains for GT-B fold glycosyltransferases create a cleft for acceptor binding while the B domain contains the active site for catalysis. B3GLCT, a GT-A fold glycosyltransferase, adopts a similar domain structure as POFUT1 and POFUT2 and other GT-B fold glycosyltransferases. A domains are colored grey, B domains are colored cyan and TSRs or EGFs are colored pink.

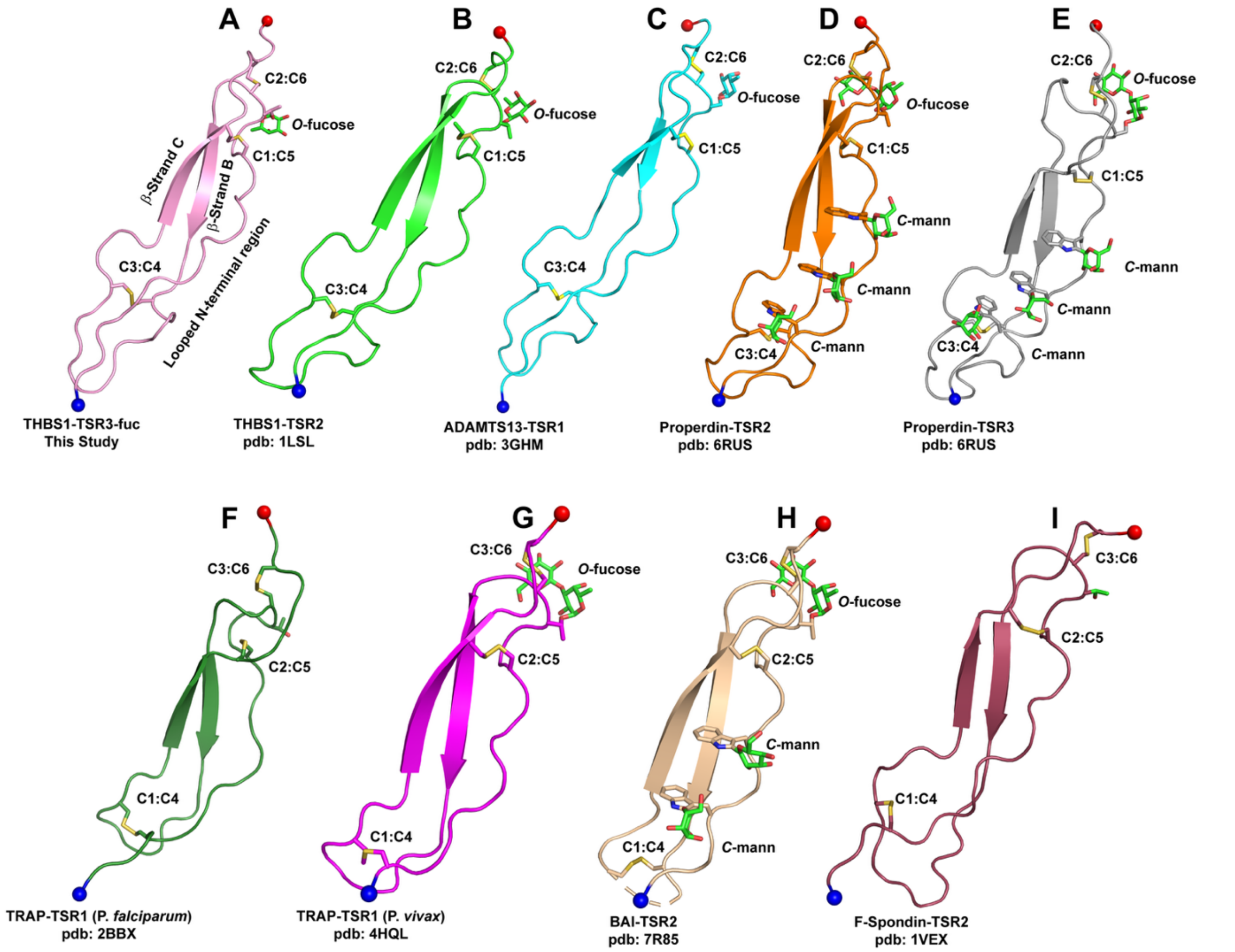

Figure S5: Similar three-dimensional structures of multiple Group 1 and Group 2 TSRs. The structures of many TSRs shows they have similar structures and shape. (A) The cartoon representation of Fuc-*O*-TSR3 from this study. Fuc-*O*-TSR3 is composed of an N-terminal A strand followed by β-strands B1 and B2, that form an antiparallel β-sheet. Each strand is connected by a small loop; and the antiparallel β-sheet in each TSR can be short (C) or stretch almost the entire length of the TSR (H). Fuc-*O*-TSR3 is categorized as a Group 1 TSR due to its C1:C5, C2:C6, C3:C4 disulfide bond configuration with the *O*-fucosylation consensus sequence occurring between C1 and C2. Group 2 TSRs form a C1:C4, C2:C5, C3:C6 disulfide bond configuration with the *O*-fucosylation consensus sequence occurring between C2 and C3 (F). Both Group 1 and Group 2 TSRs can be *C*-mannosylated if they contain the [W-X-X-(W/C)] consensus sequence (D-E, H). (A-E) are Group 1 TSRs and (F-I) are Group 2 TSRs. (B) TSR2 from THBS1 (7). (C) TSR1 from ADAMTS13 (8). (D) TSR2 from Properdin (9). (E) TSR3 from Properdin (9). (F) TSR1 from *P. falciparum* TRAP protein (10). (G) TSR1 from *P. vivax* TRAP protein (11). (H) TSR2 from BAI (12). (I) TSR2 from F-Spondin (13).

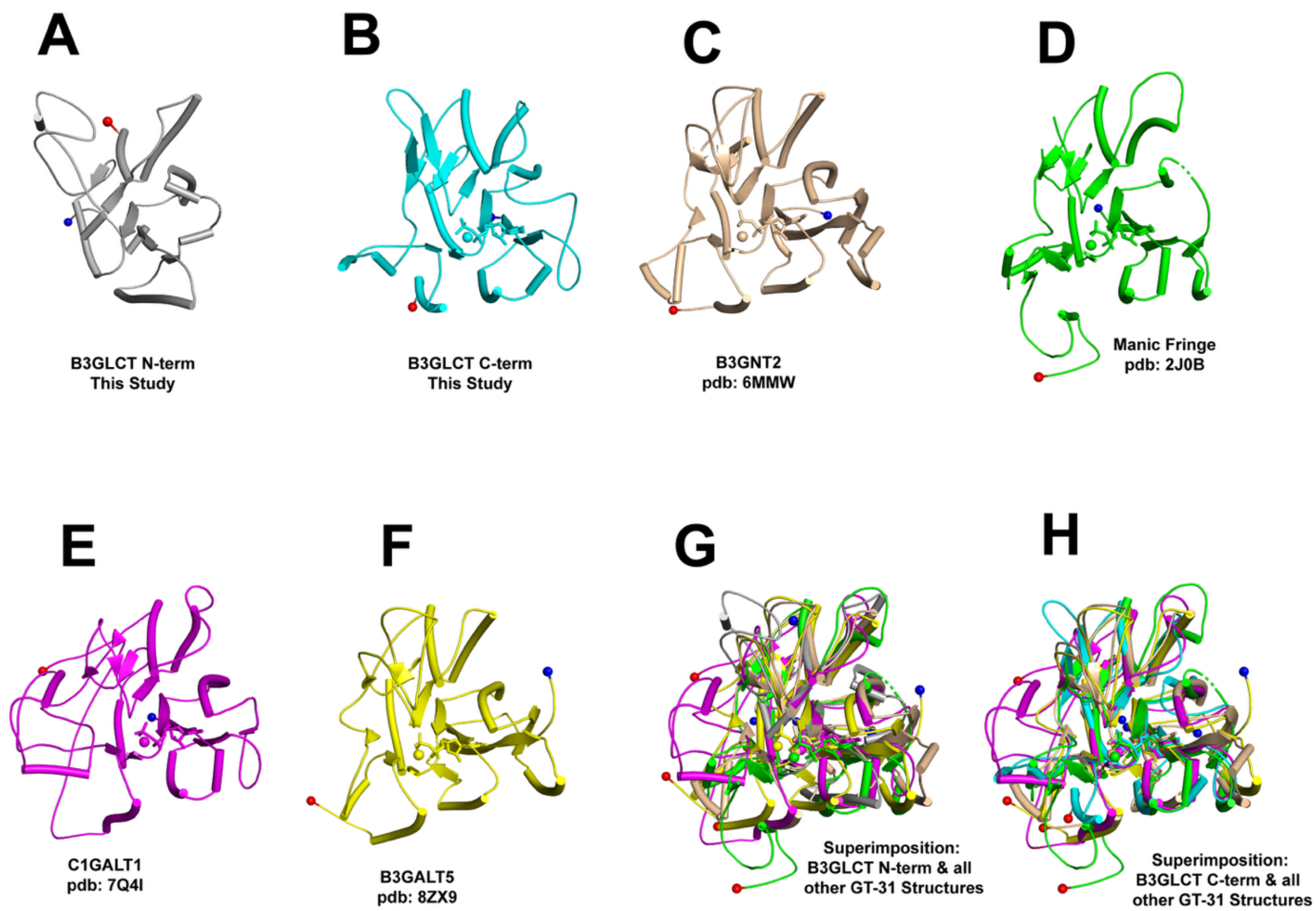

Figure S6: Superimposition of other GT-31 structures onto B3GLCT N- and C-terminal domains. (A) B3GLCT N-terminal domain from this study. (B) Catalytic B3GLCT C-terminal domain from this study. (C) GT-31 family member B3GNT2 (14). (D) GT-31 family member Manic Fringe (6). (E) GT-31 family member C1GALT1 (15). (F) GT-31 family member B3GALT5 (16). (G) Superimposition of structures (C-F) onto the B3GLCT N-term from this study (A) to highlight the structural similarity of the B3GLCT N-terminal domain and other GT-31 family members. (H) Superimposition of structures (C-F) onto the B3GLCT C-term from this study (B) to highlight the structural similarity of the B3GLCT C-terminal domain and other GT-31 family members. Superimpositions were performed using PyMOL™ (v.3.1).

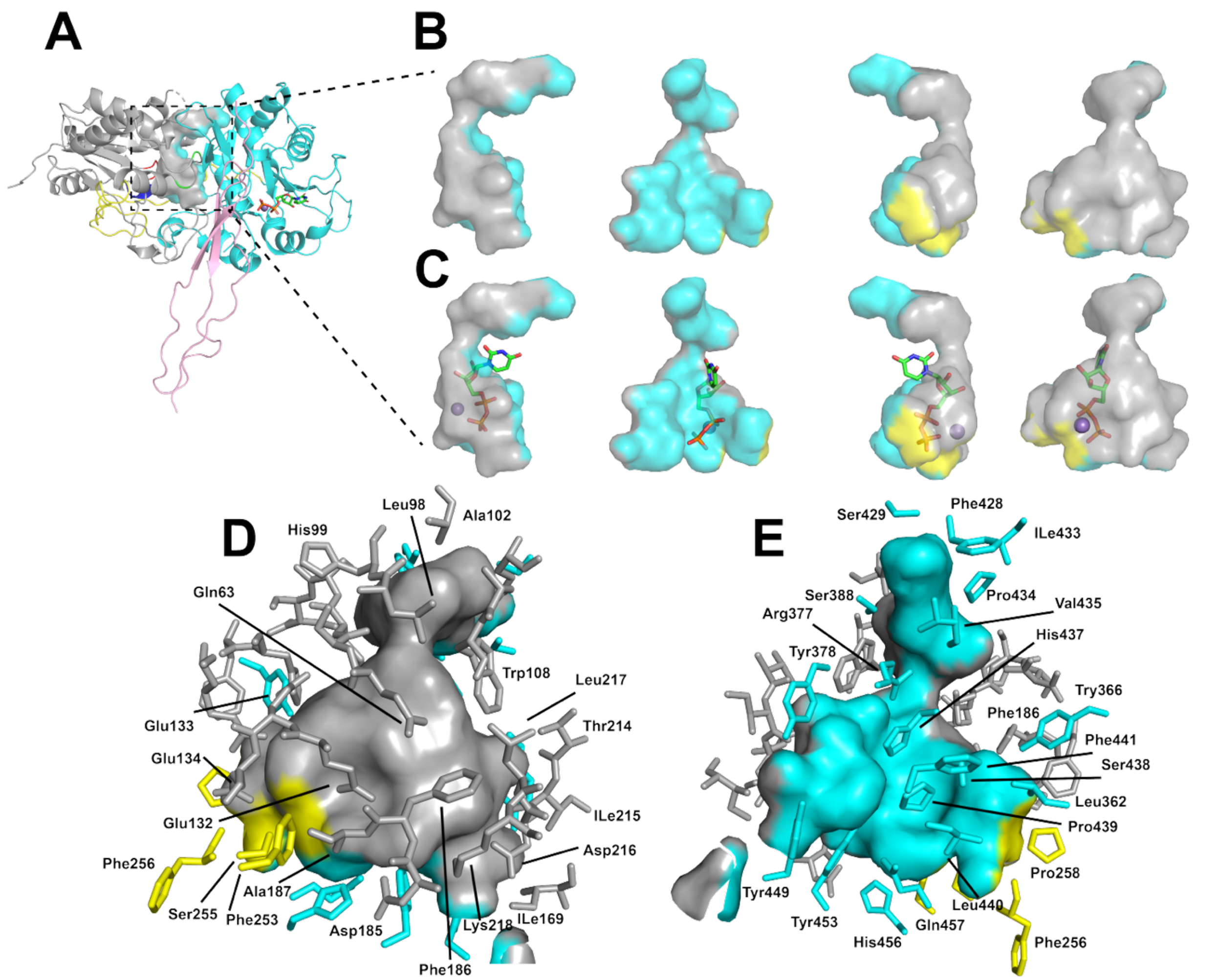

Figure S7: The N- and C-terminal Domains create a deep pocket near the N-terminal vestigial active site. (A) Overall crystal structure of Fuc-*O*-TSR3 highlighting the location of the deep pocket located between the N-terminal and C-terminal domains. (B) A closeup view of the deep pocket and subsequently being turned 90° for each of the following three cavity images. (C) A closeup view of the deep pocket showing the UDP and Mn^{2+} from the C-terminal domain and how it fits into the cavity when superimposed onto the N-terminal domain. (D) Residues from the N-terminal domain and the linker region that cradle and shape the deep pocket. (E) Residues from the C-terminal domain and the linker region that cradle and shape the deep pocket. Grey, residues from the N-terminal domain; cyan, residues from the C-terminal domain; yellow, residues from the linker region.

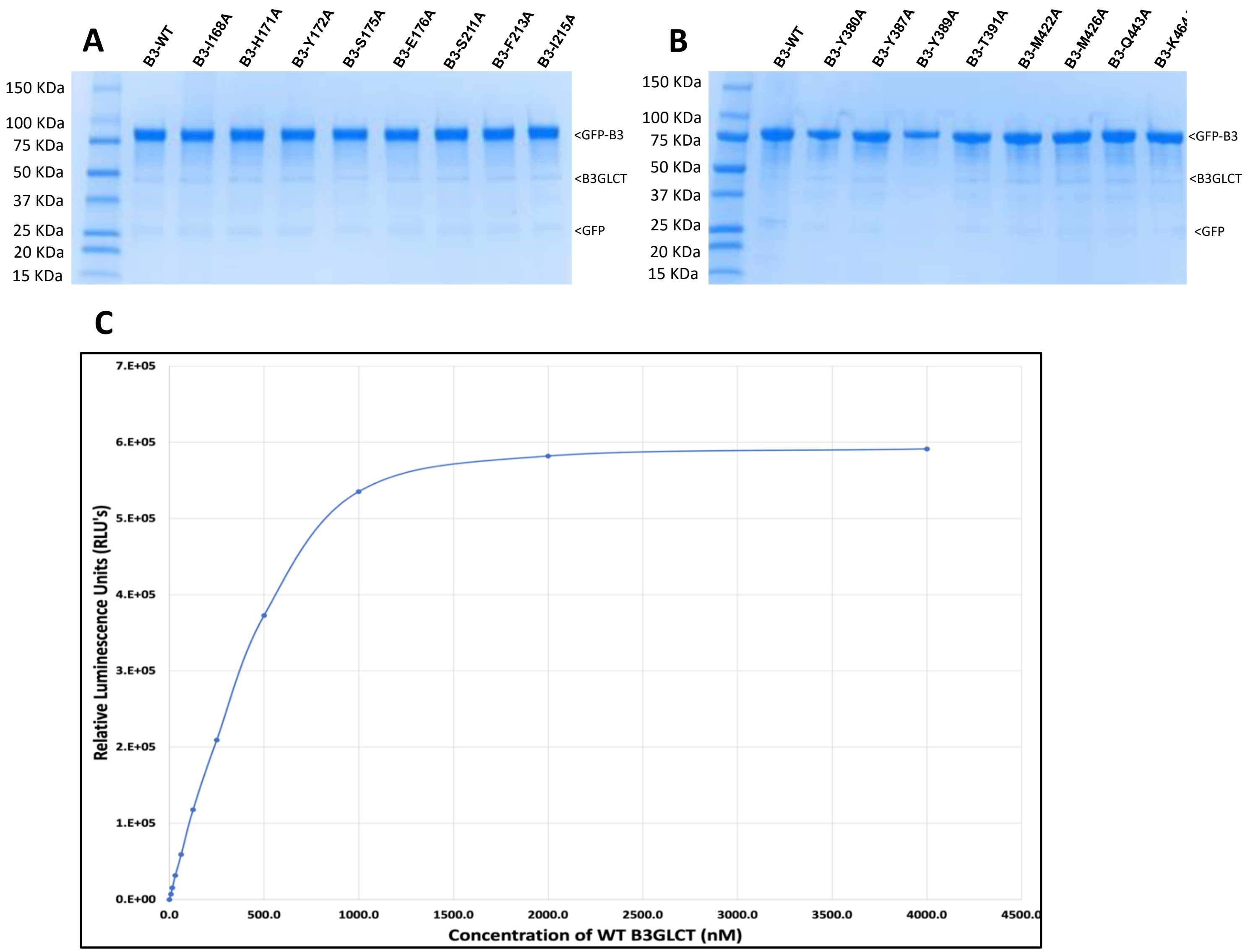

Figure S8: Expression of B3GLCT mutants and determination of linear range of B3GLCT kinetics. (A) After Ni-NTA and size exclusion chromatography purification, Coomassie stained SDS-PAGE shows the purity of wild type B3GLCT and B3GLCT N-terminal domain mutants used for kinetic experiments. (B) Coomassie stained SDS-PAGE shows the purity of wild type B3GLCT and B3GLCT C-terminal domain mutants. The wild type B3GLCT in lane 2 on both gels is from the same purification. (C) To determine the linear range of wild type B3GLCT, reactions were saturated with Fuc-*O*-TSR3 acceptor and UDP-Glucose donor B3GLCT. A concentration of 600 nM of B3GLCT was used for substrate saturation curves. The linear range was determined by a single replicate.

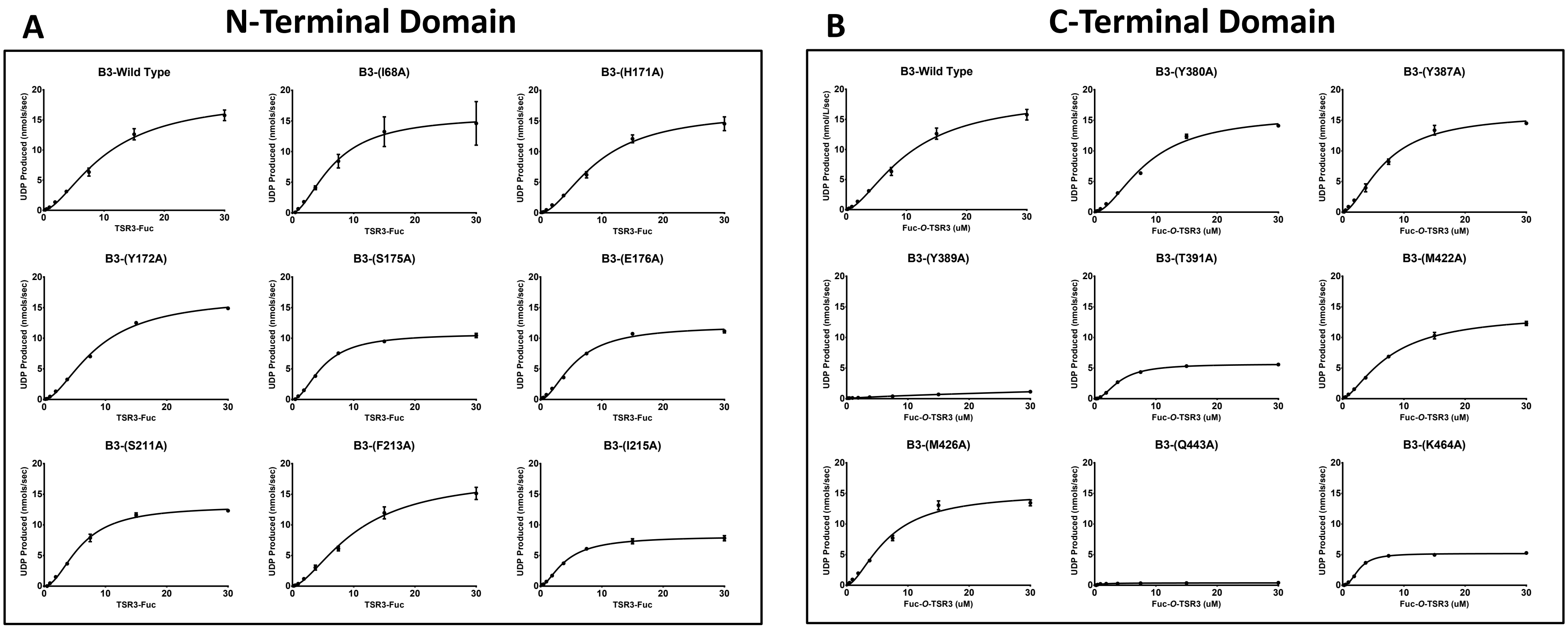

Figure S9A-B: B3GLCT mutants substrate saturation curves with varying Fuc-*O*-TSR3 acceptor. (A) Substrate saturation curves for N-terminal domain mutants. (B) Substrate saturation curves for C-terminal domain mutants. The curves shown above and data presented in Table S5 were generated in GraphPad Prism and were fit using allosteric sigmoidal least squares fitting. Residues mutated in the N-terminal domain (A) did not show significant changes in kinetic parameters when compared to wild type. Multiple residues mutated in the C-terminal domain (B3-Y389A, B3-Q443A and B3-K464A), showed a significant effect on kinetic parameters when compared to wild type. Wild type curves in both (A) and (B) are the same data and fit. All substrate saturation curves are technical triplicates. Errors bars for each substrate saturation curve are reported as standard deviation of the mean and were calculated in GraphPad Prism v.6.01 (Siemens).

C **N-Terminal Domain**

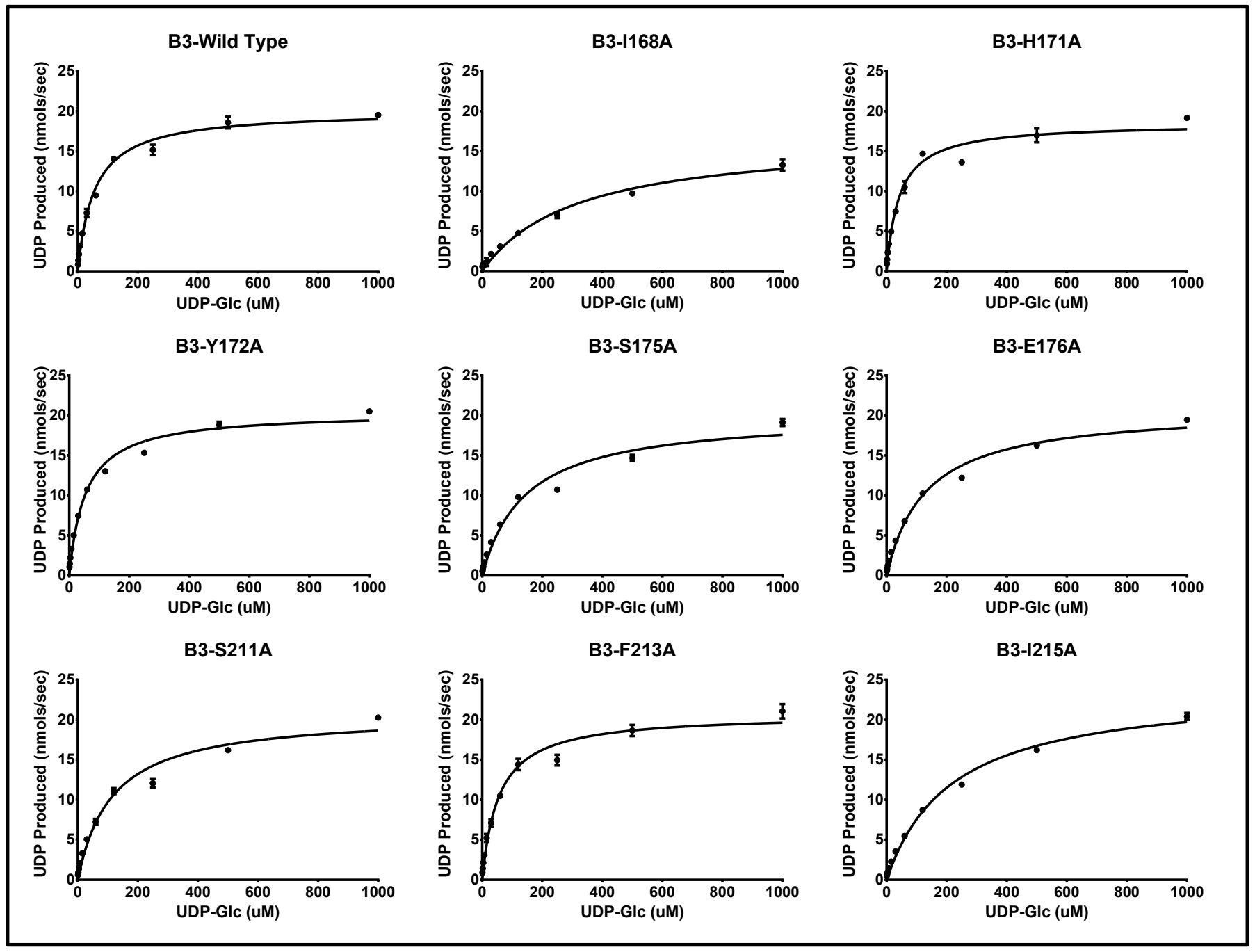

D **C-Terminal Domain**

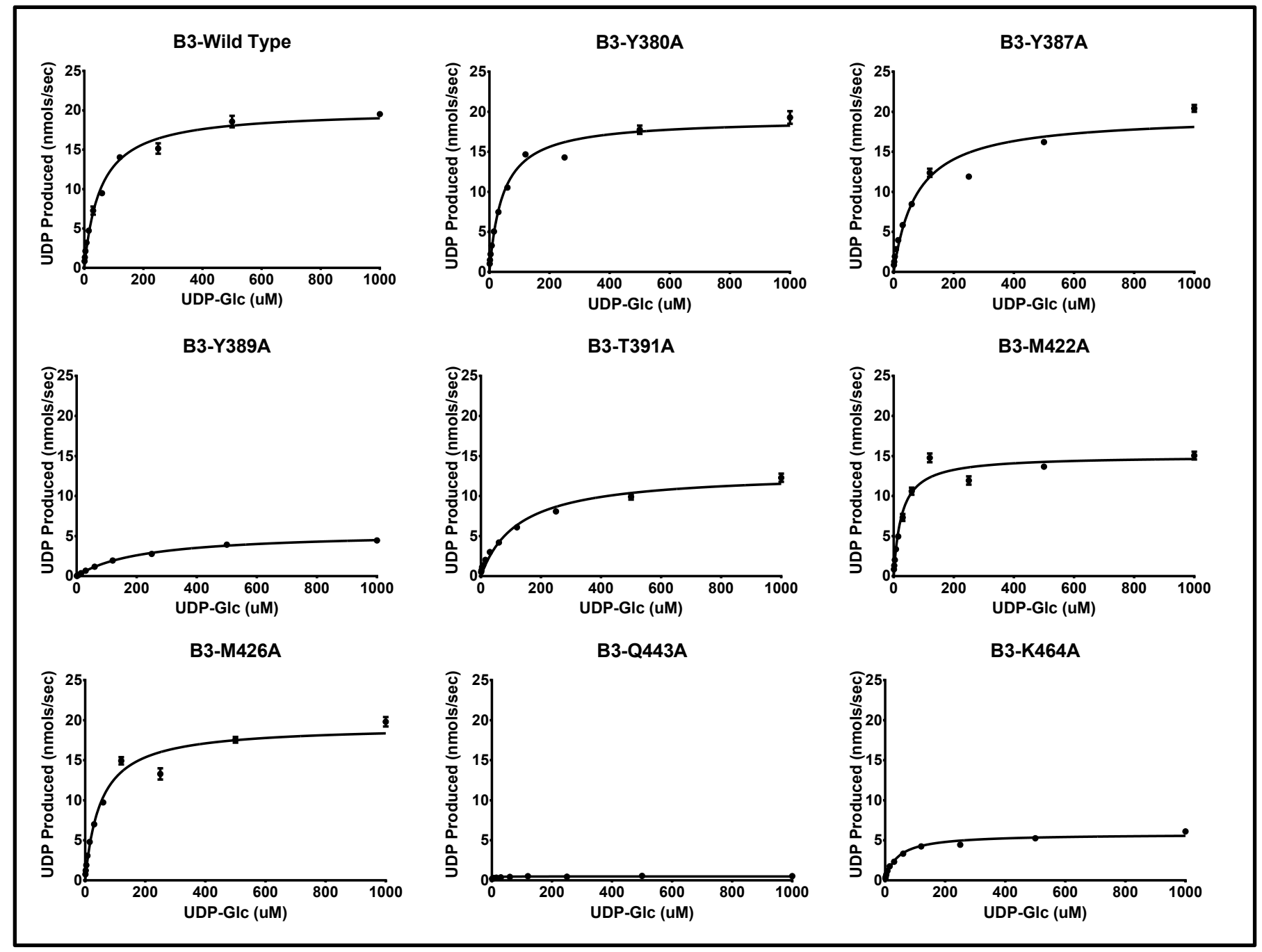

Figure S9C-D: B3GLCT mutants substrate saturation curves with varying UDP-Glucose donor. (A) Substrate saturation curves for N-terminal domain mutants. (B) Substrate saturation curves for C-terminal domain mutants. The curves shown above and data presented in Table S5 were generated in GraphPad Prism and were subjected to Michaelis-Menten least squares fitting. Residues mutated in the N-terminal domain (A) did not show a significant effect on kinetic parameters when compared to wild type. Multiple residues mutated in the C-terminal domain (B3-Y389A, B3-Q443A and B3-K464A), showed a significant effect on kinetic parameters when compared to wild type. Wild type curves in both (A) and (B) are the same data and fit. All substrate saturation curves are technical triplicates. Errors bars for each substrate saturation curve are reported as standard deviation of the mean and were calculated in GraphPad Prism v.6.01 (Siemens).

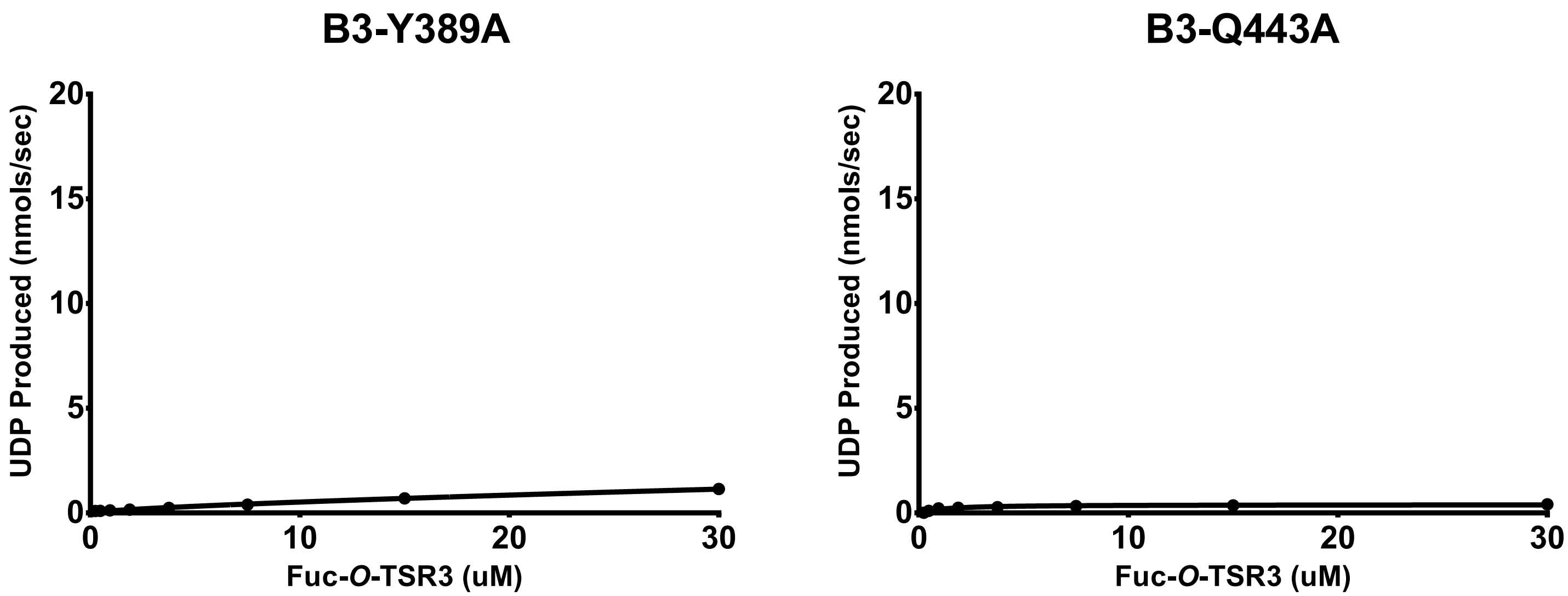

Figure S9E: Zoom in of substrate saturation curves varying Fuc-*O*-TSR3 for B3 mutants Y389A and Q443A. Both mutants display nonsaturable kinetics for the Fuc-*O*-TSR3 acceptor. All substrate saturation curves are technical triplicates. Errors bars for each substrate saturation curve are reported as standard deviation of the mean and were calculated in GraphPad Prism v.6.01 (Siemens).

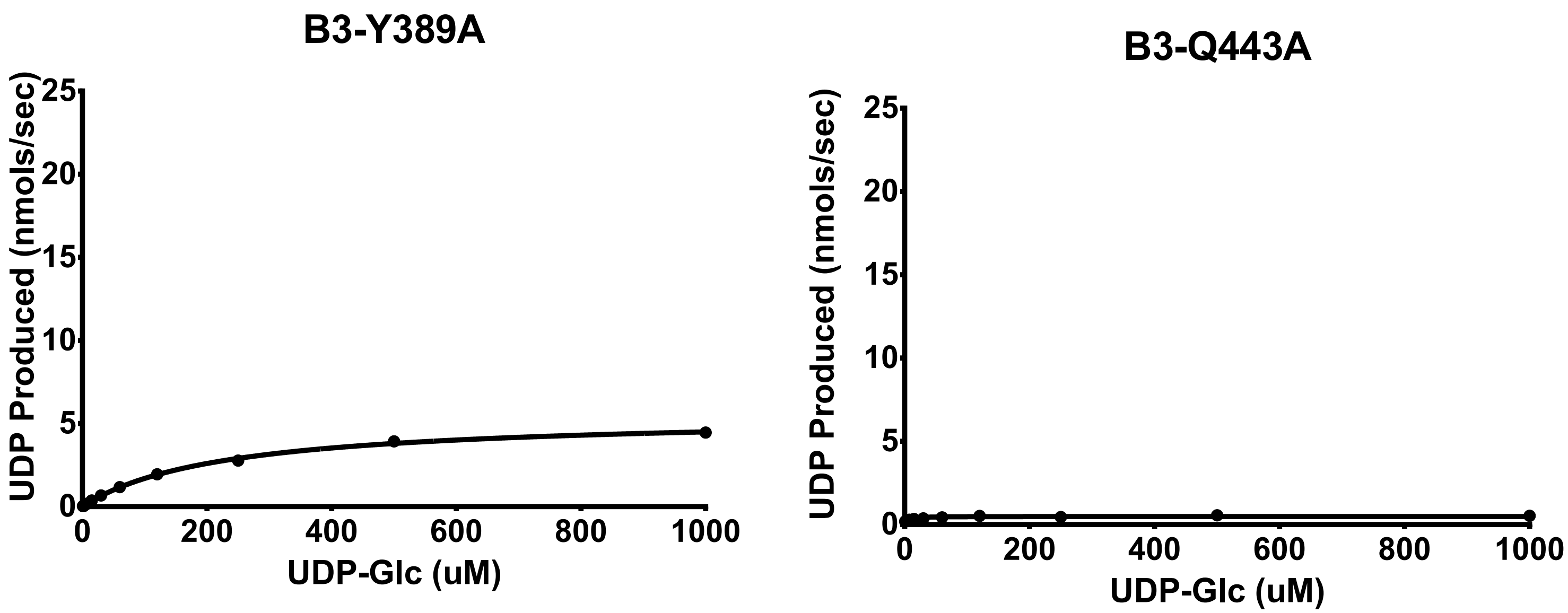

Figure S9F: Zoom in of substrate saturation curves varying UDP-glucose for B3 mutants Y389A and-Q443A. Both mutants display nonsaturable kinetics for the UDP-Glucose donor. All substrate saturation curves are technical triplicates. Errors bars for each substrate saturation curve are reported as standard deviation of the mean and were calculated in GraphPad Prism v.6.01 (Siemens).

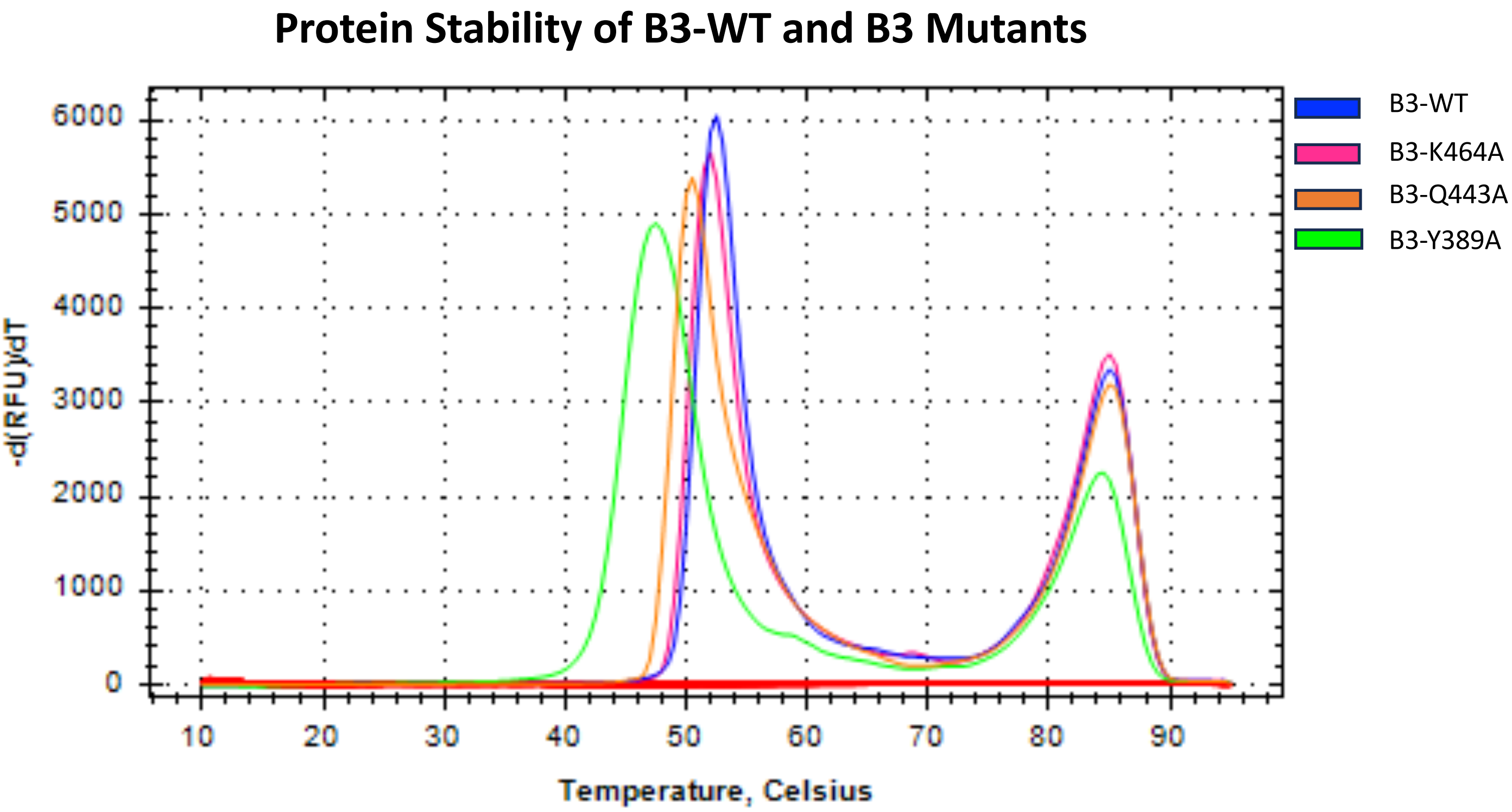

Figure S10: Protein stability assays of B3GLCT mutants with poor activity. Thermal denaturation curves of B3-WT and B3 mutants that had decreased activity. B3-WT and B3-K464 became denatured at roughly 52 °C. B3-Q443A denatured 1 degree earlier than B3-WT while B3-Y389A showed a significant decrease in stability, denaturing at 46 °C. All Thermal denaturation curves are a single replicate and denaturation temperatures were determined by eye due to ease of interpretation.

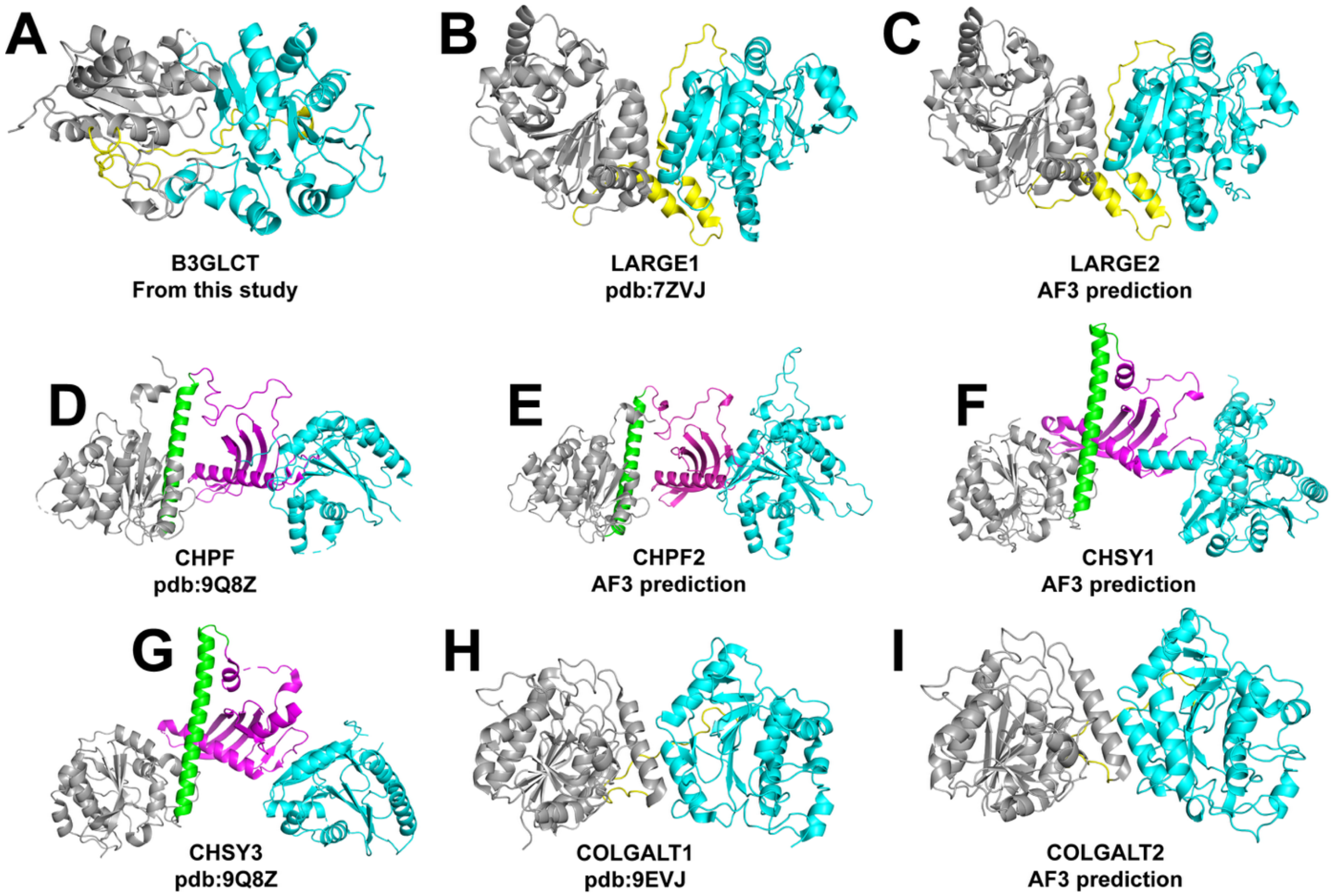

Figure S11: B3GLCT and the structural comparison with all other human two GT-A domain containing enzymes. (A) B3GLCT from this study. (B) Crystal structure of LARGE1 (17). (C) AlphaFold3 prediction of LARGE2 structure. (D) Cryo-EM structure of CHFP (18). (E) AlphaFold3 prediction of CHPF2 structure. (F) AlphaFold3 prediction of CHSY1 structure. (G) Structure of CHSY3 (18). (H) Structure of COLGALT1 (19). (I) AlphaFold3 prediction of COLGALT2 structure. For all images in this figure: N-terminal domains are colored in grey. C-terminal domains are colored in cyan. Linker regions are colored in yellow. Large alpha-helices and cystatin-like domains in chondroitin sulfate synthesis enzymes are colored green and magenta, respectively.

References for Supporting Information

1. Diederichs, K., and Karplus, P. A. (1997) Improved R-factors for diffraction data analysis in macromolecular crystallography *Nat Struct Biol* **4**, 269-275 10.1038/nsb0497-269
2. Dassie-Ajdid, J., Causse, A., Poidvin, A., Granier, M., Kaplan, J., Burglen, L. *et al.* (2009) Novel B3GALTL mutation in Peters-plus Syndrome *Clin Genet* **76**, 490-492 10.1111/j.1399-0004.2009.01253.x
3. Weh, E., Reis, L. M., Tyler, R. C., Bick, D., Rhead, W. J., Wallace, S. *et al.* (2014) Novel B3GALTL mutations in classic Peters plus syndrome and lack of mutations in a large cohort of patients with similar phenotypes *Clin Genet* **86**, 142-148 10.1111/cge.12241
4. Zhang, A., Venkat, A., Taujale, R., Mull, J. L., Ito, A., Kannan, N. *et al.* (2021) Peters plus syndrome mutations affect the function and stability of human beta1,3-glucosyltransferase *J Biol Chem* **297**, 100843 10.1016/j.jbc.2021.100843
5. Valero-Gonzalez, J., Leonhard-Melief, C., Lira-Navarrete, E., Jimenez-Oses, G., Hernandez-Ruiz, C., Pallares, M. C. *et al.* (2016) A proactive role of water molecules in acceptor recognition by protein O-fucosyltransferase 2 *Nat Chem Biol* **12**, 240-246 10.1038/nchembio.2019
6. Jinek, M., Chen, Y. W., Clausen, H., Cohen, S. M., and Conti, E. (2006) Structural insights into the Notch-modifying glycosyltransferase Fringe *Nat Struct Mol Biol* **13**, 945-946 10.1038/nsmb1144
7. Tan, K., Duquette, M., Liu, J. H., Dong, Y., Zhang, R., Joachimiak, A. *et al.* (2002) Crystal structure of the TSP-1 type 1 repeats: a novel layered fold and its biological implication *J Cell Biol* **159**, 373-382 10.1083/jcb.200206062
8. Akiyama, M., Takeda, S., Kokame, K., Takagi, J., and Miyata, T. (2009) Crystal structures of the noncatalytic domains of ADAMTS13 reveal multiple discontinuous exosites for von Willebrand factor *Proc Natl Acad Sci U S A* **106**, 19274-19279 10.1073/pnas.0909755106
9. Pedersen, D. V., Gadeberg, T. A. F., Thomas, C., Wang, Y., Joram, N., Jensen, R. K. *et al.* (2019) Structural Basis for Properdin Oligomerization and Convertase Stimulation in the Human Complement System *Front Immunol* **10**, 2007 10.3389/fimmu.2019.02007
10. Tossavainen, H., Pihlajamaa, T., Huttunen, T. K., Raulo, E., Rauvala, H., Permi, P. *et al.* (2006) The layered fold of the TSR domain of P. falciparum TRAP contains a heparin binding site *Protein Sci* **15**, 1760-1768 10.1110/ps.052068506
11. Song, G., Koksal, A. C., Lu, C., and Springer, T. A. (2012) Shape change in the receptor for gliding motility in Plasmodium sporozoites *Proc Natl Acad Sci U S A* **109**, 21420-21425 10.1073/pnas.1218581109
12. Wang, J., Miao, Y., Wicklein, R., Sun, Z., Wang, J., Jude, K. M. *et al.* (2022) RTN4/NoGo-receptor binding to BAI adhesion-GPCRs regulates neuronal development *Cell* **185**, 218 10.1016/j.cell.2021.12.017
13. Paakkonen, K., Tossavainen, H., Permi, P., Rakkolainen, H., Rauvala, H., Raulo, E. *et al.* (2006) Solution structures of the first and fourth TSR domains of F-spondin *Proteins* **64**, 665-672 10.1002/prot.21030
14. Kadirvelraj, R., Yang, J. Y., Kim, H. W., Sanders, J. H., Moremen, K. W., and Wood, Z. A. (2021) Comparison of human poly-N-acetyl-lactosamine synthase structure with GT-A

fold glycosyltransferases supports a modular assembly of catalytic subsites *J Biol Chem* **296**, 100110 10.1074/jbc.RA120.015305
15. Gonzalez-Ramirez, A. M., Grosso, A. S., Yang, Z., Companon, I., Coelho, H., Narimatsu, Y. *et al.* (2022) Structural basis for the synthesis of the core 1 structure by C1GalT1 *Nat Commun* **13**, 2398 10.1038/s41467-022-29833-0
16. Lo, J. M., Kung, C. C., Cheng, T. R., Wong, C. H., and Ma, C. (2025) Structure-Based Mechanism and Specificity of Human Galactosyltransferase beta3GalT5 *J Am Chem Soc* **147**, 10875-10885 10.1021/jacs.4c11724
17. Katz, M., and Diskin, R. (2022) Structural basis for matriglycan synthesis by the LARGE1 dual glycosyltransferase *PLoS One* **17**, e0278713 10.1371/journal.pone.0278713
18. Dutta, P., Cordeiro, R. L., Friedel-Arboleas, M., Bourgeais, M., Vallet, S. D., Weber, M. *et al.* (2025) Structural basis for human chondroitin sulfate chain polymerization *Nat Commun* 10.1038/s41467-025-66787-5
19. De Marco, M., Rai, S. R., Scietti, L., Mattoteia, D., Liberi, S., Moroni, E. *et al.* (2025) Molecular structure and enzymatic mechanism of the human collagen hydroxylysine galactosyltransferase GLT25D1/COLGALT1 *Nat Commun* **16**, 3624 10.1038/s41467-025-59017-5
